# Chemical Tools for Probing the Ub/Ubl Conjugation Cascades

**DOI:** 10.1002/cbic.202400659

**Published:** 2024-11-06

**Authors:** Tomasz Kochańczyk, Michael Fishman, Christopher D. Lima

**Affiliations:** ^1^ Structural Biology Program Sloan Kettering Institute 1275 York Avenue New York, New York 10065 USA; ^2^ Howard Hughes Medical Institute 1275 York Avenue New York, New York 10065 USA

**Keywords:** Ubiquitin, Ubiquitin-like protein, E1, E2, E3 ligase

## Abstract

Conjugation of ubiquitin (Ub) and structurally related ubiquitin‐like proteins (Ubls), essential for many cellular processes, employs multi‐step reactions orchestrated by specific E1, E2 and E3 enzymes. The E1 enzyme activates the Ub/Ubl C‐terminus in an ATP‐dependent process that results in the formation of a thioester linkage with the E1 active site cysteine. The thioester‐activated Ub/Ubl is transferred to the active site of an E2 enzyme which then interacts with an E3 enzyme to promote conjugation to the target substrate. The E1‐E2‐E3 enzymatic cascades utilize labile intermediates, extensive conformational changes, and vast combinatorial diversity of short‐lived protein‐protein complexes to conjugate Ub/Ubl to various substrates in a regulated manner. In this review, we discuss various chemical tools and methods used to study the consecutive steps of Ub/Ubl activation and conjugation, which are often too elusive for direct studies. We focus on methods developed to probe enzymatic activities and capture and characterize stable mimics of the transient intermediates and transition states, thereby providing insights into fundamental mechanisms in the Ub/Ubl conjugation pathways.

## Graphical abstract

Post‐translational modification by ubiquitin (Ub) and ubiquitin‐like proteins (Ubl) regulate a diverse array of cellular pathways. Ub/Ubl conjugation is orchestrated by a cascade of specialized E1, E2, and E3 enzymes. Here, we review chemical tools and methods implemented to probe enzymatic activities and to capture and characterize fleeting chemical and conformational states within Ub and Ubl conjugation cascades.

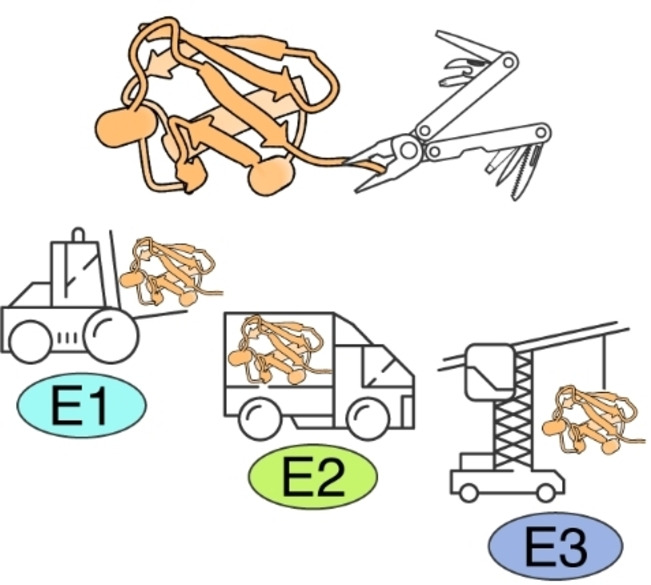

## 1 Introduction

Conjugation of ubiquitin (Ub) and related ubiquitin‐like proteins (Ubls) plays important roles in cellular processes by modulating the activity, subcellular localization, or fate of targeted substrates.^[1]^ Ub and Ubls are most often conjugated through an isopeptide bond formed between the Ub/Ubl C‐terminal carboxyl group and the amino group of lysine residue side chains within protein substrates. In recent years, alternative substrates such as lipids, sugars, and nucleotides, as well as alternative linkage types such as ester bonds have also been identified.^[2]^

**Figure 1:**
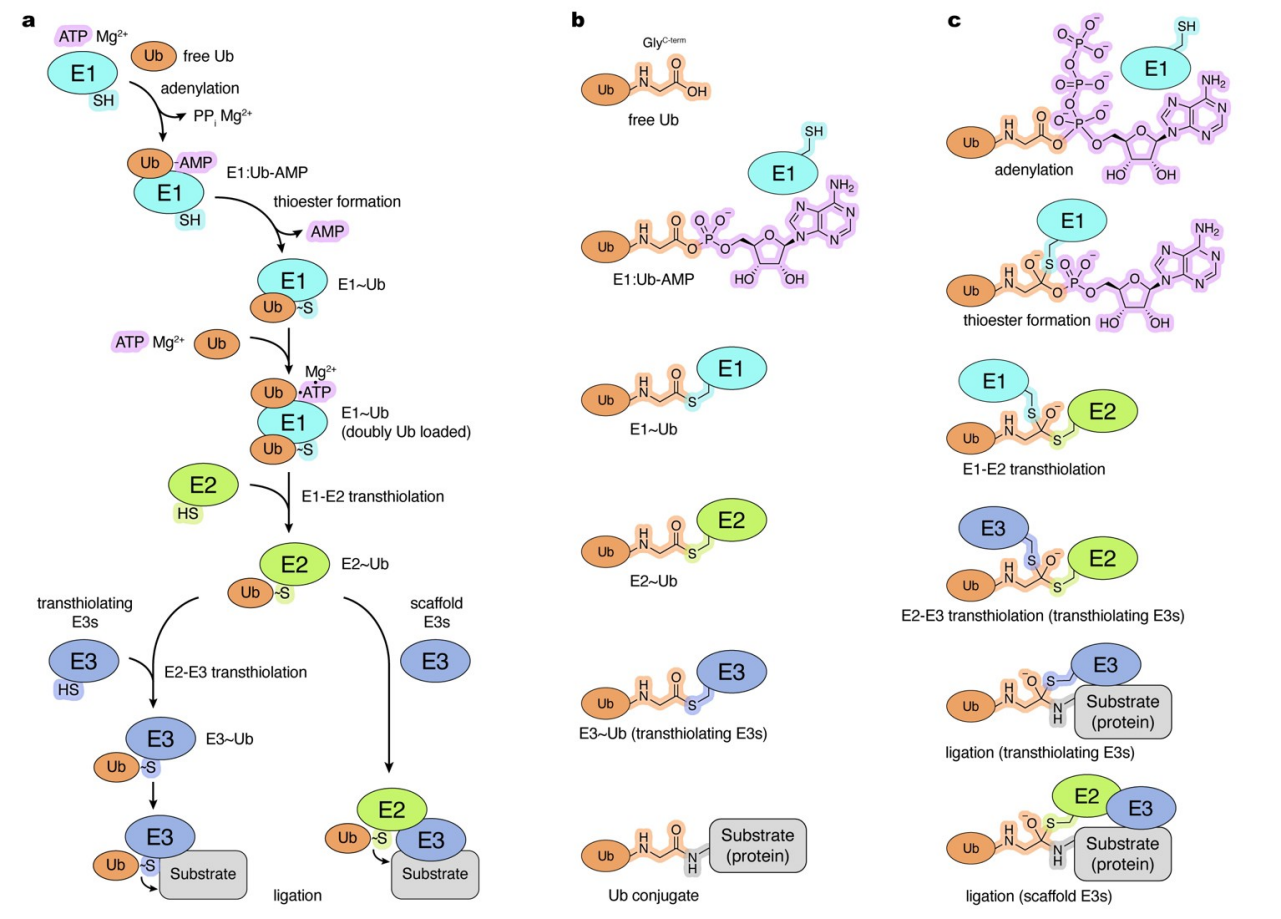
Ub conjugation cascade. a, Ub conjugation involves a series of reactions catalyzed by E1 enzymes (two found in the human proteome), E2 enzymes (approximately 35 in the human proteome), and E3 enzymes (approximately 600 in the human proteome). Distinct mechanisms for the two groups of E3 ligases (transthiolating and scaffold‐type) are shown. Tilde (~) denotes thioester bond between the C‐terminal glycine of Ub and thiol of active site cysteine; b, Ub reactants and final product (Ub conjugate) of the consecutive reactions in the Ub conjugation cascade. c, Transition states of the consecutive reactions in the Ub conjugation cascade. Mg^2+^ ions required for adenylation are not shown for clarity. Schematic and chemical representations are color‐coded and labelled.

Activation and conjugation of Ub and Ubls such as SUMO, NEDD8, ATG8, ATG12, UFM1, FAT10, and ISG15 is orchestrated by dedicated enzymatic cascades consisting of E1 activating enzymes, E2 conjugating enzymes, and in most instances, E3 ligases.[^3^, ^4^, ^5^] The consecutive reactions and enzymes are best characterized for the Ub pathway (Figure 1a) but it is now apparent that Ubl pathways utilize structurally and functionally related enzymes and some enzymes can function in multiple pathways.[^3^, ^4^] The E1 enzymes activate their cognate Ub/Ubl through formation of a C‐terminal acyl adenylate (Ub/Ubl‐AMP) in an ATP‐dependent manner. In the second step, the resulting Ub/Ubl‐AMP product undergoes a nucleophilic attack by the active‐site cysteine of the E1 enzyme, forming an E1~Ub/Ubl complex linked through a thioester bond (hereafter denoted as ~) and releasing AMP. The E1~Ub and E1~Ubl complexes for Ubls such as NEDD4, SUMO and ISG15 have been shown to undergo a subsequent round of ATP and Ub/Ubl binding at the adenylation site to form a doubly loaded complex. Finally, the E1~Ub/Ubl interacts with one of the E2 enzymes to transfer its C‐terminally thioester‐linked Ub/Ubl to the E2 active site cysteine through an E1‐E2 transthiolation (transthioesterification) reaction. The resulting E2~Ub/Ubl can then interact with an E3 ligase to facilitate covalent attachment of Ub/Ubl to the target substrate.

E3 ligases dictate substrate specificity and constitute the most structurally and mechanistically diverse group of enzymes compared to E1 and E2. The majority of Ub/Ubl E3 ligases, including many hundreds of RING (really interesting new gene) E3s function as molecular scaffolds, simultaneously binding to substrate and E2~Ub/Ubl in a catalytically competent conformation primed for conjugation.^[6]^ In contrast, transthiolating E3s include more than two dozen HECT (homologous to E6AP C‐terminus) E3s,^[7]^ over a dozen RING‐between‐RING (RBR) E3s,^[8]^ and the RING‐Cys‐Relay (RCR) E3 as exemplified by the E3 ligase MYCBP2.^[9]^ These E3s first transfer Ub/Ubl from an E2~Ub/Ubl to their active site cysteine through E2‐E3 transthiolation to form an E3~Ub/Ubl complex as an intermediate preceding Ub/Ubl conjugation to substrates.

E3 enzymes can generate both linear and branched Ub/Ubl polymeric chains through multiple rounds of conjugation cycles, thereby increasing the complexity of elicited Ub/Ubl signals.[^1^, ^10^, ^11^] These signals are recognized by various cellular effectors, leading to specific functional responses. The covalent attachment of Ub/Ubl can be reversed by deubiquitinating enzymes (DUBs) and Ubl‐specific proteases (ULPs).^[12]^ Thus, the Ub/Ubl signaling is not only remarkably diverse but also highly dynamic.

Identifying components and characterizing consecutive steps in Ub/Ubl activation and conjugation cascades is crucial for understanding the molecular mechanisms and, ultimately, biological functions of these pathways. This has proven to be challenging, as Ub/Ubl activation and conjugation proceed through formation and rapid dissolution of transient complexes and reaction intermediates (Figure 1b,c). These transient states exist at low steady‐state levels and are often too chemically labile or technically difficult to isolate and examine directly. Transition states are perhaps the most challenging, as they represent the highest free energy structures along the reaction coordinate between reactants and products, with a correspondingly strained configuration and extremely short lifetime.^[13]^ Because of the fleeting nature of these states, a frequent strategy is to employ chemical tools such as activity‐based probes, inhibitors, or non‐hydrolysable substrates to obtain stable analogues of reaction intermediates and transition states to investigate their mechanistic relevance. These tools facilitated structure determination of intermediates that were not observed previously, providing insights into functionally important interactions and how amino acid substitutions may affect protein function and potentially cause disease. Furthermore, these observations provide opportunities for pharmacological intervention through development of allosteric inhibitors or activators, or by stabilizing specific conformational states to capture interactions between conjugation enzymes and their target substrates.

The application of chemical tools that selectively interact with or react with components of the conjugation machinery has been instrumental in advancing our understanding of Ub/Ubl pathways. Here, we review chemical tools used in studies of Ub/Ubl conjugation cascades focusing on approaches used to probe enzyme activities and characterize fleeting reaction intermediates and transition states. We begin by discussing common types of chemical tools used to study Ub/Ubl cascades and their desirable properties, focusing on the broadly used activity‐based probes. We then review work introducing probes exhibiting broad reactivity towards enzymes in the Ub/Ubl pathways and laying the groundwork for numerous subsequent strategies. Following this, we introduce more specific examples of chemical tools categorized by their utility in studying specific steps of Ub/Ubl activation and conjugation. The discussion is focused on highlighting the methods that led to landmark discoveries or were important for the development of other chemical tools. Additionally, the advantages and limitations of each strategy are discussed. In the last section we discuss the challenges and further potential for chemical tools in studies of the Ub/Ubl pathways.

## 2 General Characteristics of Activity‐Based Probes and Other Strategies

Our understanding of Ub/Ubl pathways has been advanced by implementing synthetic or semi‐synthetic molecules to target functional states of mechanistically related enzymes through common interaction patterns and active site chemistry. These molecules, referred to as activity‐based probes (ABPs), typically form covalent bonds with enzyme active sites. ABPs are often defined as probes with sufficient specificity and reactivity to native enzyme activities to enable functional profiling of enzymes in whole proteomes. Here, we use this term more broadly, referring to all probes that leverage molecular recognition or react with enzymes, including those that found usage in less complex biological settings, such as with purified enzymes.

**Figure 2:**
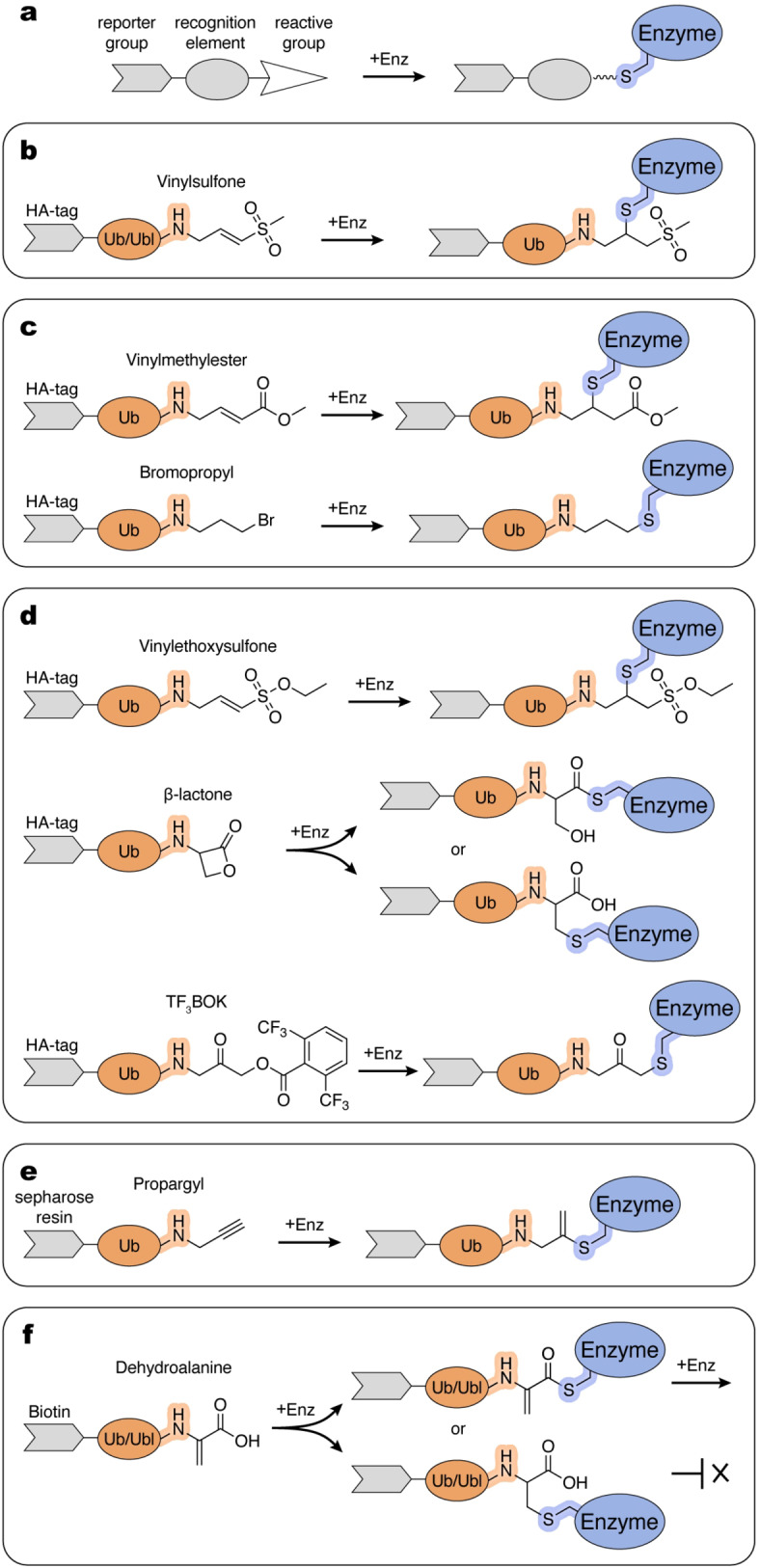
ABPs used to study Ub/Ubl (de)conjugation enzymes. a, General architecture of ABPs. b–f, Examples of the thiol‐reactive Ub/Ubl‐derived ABPs and possible modes of reactivity with enzyme active sites. Schematic and chemical representations are color‐coded and labelled. Atoms and linkages corresponding to the amino group of the penultimate Gly (Gly^−1^) of the Ub/Ubl and active site cysteine of an enzyme are highlighted in orange and blue, respectively.

Most ABPs share a similar modular architecture consisting of: (i) a reactive group that forms a covalent bond with the enzyme, (ii) a recognition element that confers specific binding, and (iii) a reporter group used for detection (Figure 2a).^[14]^

The reactive group (i), sometimes referred to as the “warhead”, typically displays some degree of specific reactivity towards active site chemistry. Electrophilic moieties are often utilized due to their reactivity with nucleophilic thiols of cysteines present in the active sites of many enzymes in Ub/Ubl pathways. Given the abundance of other competing nucleophiles, the reactivity and selectivity tend to be inversely correlated. Thus, the chemistry of reactive groups is generally tuned to minimize formation of off‐target products while maintaining sufficient reactivity with the target sites. The chemical properties of the product of the reaction, including its stability, are also important. In certain scenarios, the reactive group is engineered to generate a reaction product that resembles the bona fide intermediate as closely as possible. Such chemically tailored design is crucial if the product of the reaction is intended to be recognized by other components of the cascade or to serve as a mimic of a transition state for structural studies. The reactive group is coupled with a recognition element (ii), typically a protein, to enhance specificity and product characteristics by mimicking interactions utilized by the conjugation machinery. As most enzymatic steps utilizing Ub/Ubl proteins involve their C‐terminus, many probes contain Ub/Ubl molecules linked to reactive groups that replace or are near the C‐terminal residue of the respective Ub/Ubl. Selectivity can be modified within the protein by introducing additional elements, such as phosphorylated residues, or by inclusion of other protein components (for example, E2) to specifically target functional states involving E1 or E3 enzymes. Lastly, a reporter group (iii) is frequently incorporated into the probe to facilitate detection and/or isolation of the reaction product. Common examples include fluorophores used for detection and/or an affinity tag for purification purposes.

### 2.1 Other Strategies

Some chemical probes employed in studies of Ub/Ubl conjugation cascades do not react with active sites, but instead function by covalently modifying other regions, for example, by using photoactivatable crosslinking groups. Furthermore, certain probes have been designed to associate with the conjugation machinery exclusively via non‐covalent interactions. Notable examples of such probes are the non‐reactive analogues of tightly bound substrates, such as the adenylated Ub/Ubl molecules. Although their modes of action differ, these probes can be dissected into similar functional elements as those described for traditional activity‐based probes.

Beyond chemical probes, many other chemical strategies have been employed in studies of Ub/Ubl conjugation cascades. Various chemical reagents that can manipulate structure in a predictable manner have been used to study structure‐function relationships. Examples include reagents used to introduce site‐specific disulfide bonds, or crosslinkers used to capture stable mimics of enzyme complexes for structural analyses. These reagents are usually broadly reactive and are often applied to isolated enzymes with amino acid substitutions to remove other cysteines beyond the active site that could potentially react. Lastly, some strategies rely on introducing amino acid substitutions only. Examples include substituting active site cysteine with serine or lysine to generate more stable ester or isopeptide analogues of the thioester intermediate.

Many strategies can be envisioned to target a particular step in Ub/Ubl conjugation cascades, but to evaluate the utility of a given strategy, one must consider its synthetic feasibility, compatibility of the strategy with in vivo studies, and adaptability of the approach to other biological questions or experimental settings.

## 3 Thiol‐Reactive Ub/Ubl‐Derived ABPs

E1, E2, and certain E3 enzymes in Ub/Ubl conjugation cascades utilize an active site cysteine residue to form a thioester intermediate with the Ub/Ubl C‐terminus to facilitate transfer of the activated Ub/Ubl (Figure 1). Many deconjugating enzymes also rely on a reactive cysteine residue in their catalytic triad that forms a thioester intermediate prior to release of the product. This property has been leveraged to construct Ub/Ubl‐derived ABPs harboring a C‐terminal thiol‐reactive group that forms a stable adduct with the active site of (de)conjugating enzymes. The extent of adduct formation does not necessarily correlate with the enzyme activity. This is because ABPs that only include the Ub/Ubl moiety for recognition lack the acyl‐linked component, such as the thioester‐linked enzyme, that are typically recognized in native reactions (Figure 1).

Historically, thiol‐reactive Ub/Ubl‐derived ABPs were first introduced to study deconjugating enzymes. The first such probe, Ub‐aldehyde, was discovered serendipitously in pioneering studies by Pickart and Rose on the mechanism of the DUB ubiquitin carboxyl‐terminal hydrolase (UCHL1) nearly four decades ago.^[15]^ Since then, numerous studies have extended the range and utility of thiol‐reactive Ub/Ubl‐derived probes by utilizing new reactive groups, new methods of synthesis, and broadening their application to target enzymes in the conjugation cascades.

Borodovski and coworkers introduced a series of thiol‐reactive Ub probes functionalized with C‐terminal Michael acceptor‐derived electrophiles, such as vinylmethylsulfone (VS) and vinyl methyl ester (VME), and alkylhalide‐containing ones, such as bromopropyl (UbC3Br) (Figure 2b,c).[^16^, ^17^] They also demonstrated that an intein‐based semisynthetic method can be used to introduce chemical groups to Ub, providing a more feasible alternative to the previously used trypsin‐catalyzed transpeptidation method.^[16]^ In the intein‐based semisynthetic method, a recombinantly produced Ub lacking the C‐terminal glycine is isolated with a C‐terminal MESNa (mercaptoethanesulfonate) thioester which is used to append a reactive group.^[17]^ This method and its modifications have since been widely adopted in subsequent studies by many other research groups. Hemelaar and coworkers expanded the intein‐based chemical ligation approach to incorporate a VS group at the C‐terminus of Ubls, including NEDD8, ISG15, and SUMO1.^[18]^ While Ub/Ubl‐VS probes effectively labeled their respective DUBs and ULPs in purified form or in cell lysates, their reactivity toward enzymes that catalyze conjugation was demonstrated only using purified E1, E2, or E3 enzymes.^[18]^ The lower reactivity towards conjugation enzymes could be due to a dependance on additional recognition elements beyond the Ub/Ubl molecule, less nucleophilic active site cysteine residues or a combination of both.

To enhance reactivity towards conjugation enzymes, Love and coworkers expanded the palette of reactive Ub derivatives utilizing more electrophilic C‐terminal groups including vinylethoxysulfone (OEtVS), a β‐lactone (Lac), and a 2,6‐trifluoromethylbenzyloxymethylketone (TF_3_BOK) (Figure 2d).^[19]^ This work demonstrated the utility of the Ub‐VME, Ub‐Lac, Ub‐OEtVS, and Ub‐TF_3_BOK probes to identify components of the conjugation machinery including certain E1, E2, and E3 enzymes in cell lysates. The labeling efficiency was dependent on the specific enzyme‐ABP pair, perhaps due to differences in nucleophilicity of active site cysteine residues and their chemical environment. Of particular interest among the identified targets were the poorly characterized E3_HUWE1_ and E3_TRIP12_ HECT ligases, identified using Ub‐VME and Ub‐TF_3_BOK.^[19]^ Interestingly, HUWE1 was also isolated from cell lysates using a Ub probe containing a less electrophilic propargyl group (Ub‐Prg) introduced in a subsequent study from Ekkebus et al. (Figure 2e).^[20]^ A study comparing the reactivities of Ub‐Prg and Ub‐VME revealed differential labeling efficiencies among the purified HECT E3 ligases tested.^[21]^ Ub‐Prg reacted less efficiently with purified RBR ligase E3_HOIP_ than Ub‐VS.^[22]^ It was also shown that Ub‐Prg reacted with purified E3_HUWE1_ and E3_NEDD4_ but not with E3_E6‐AP_.^[23]^ This suggests differing structure‐activity relationships within individual active sites, indicating that a diverse array of reactive ABPs is needed to accommodate differences in active site geometries to enable profiling of diverse enzymes in Ub/Ubl pathways.

The Ub/Ubl‐derivatives described here are broadly used in mechanistic and structural studies on isolated enzymes. For example, Ub‐VME was used in studies on RBR‐type E3 ligases E3_ARIH1_ and E3_ARIH2_ to delineate their activation mechanism by binding of neddylated Cullin‐RING ligase (CRL) complexes.[^24^, ^25^] Ub‐VME was also used to obtain structural mimics of the E3_ARIH1_~Ub and HECT‐type E3_UBR5_~Ub complexes.[^26^, ^27^] Ub‐VS was used to identify a regulatory domain in the RBR‐type ligase E3_Parkin_ that inhibits its activity.[^28^, ^29^, ^30^] Parkin is stimulated by phosphorylation of Ser65 in its Ub‐like domain and the conserved Ser65 in Ub. In studies by Wauer and coworkers, Ser65‐phosphorylated Ub probes were tested for reactivity with Parkin, resulting in the discovery that a phosphor‐Ub−C3Br probe reacted with a non‐active site cysteine present in the ortholog from *Pediculus humanus*. This serendipitous discovery facilitated determination of a crystal structure that showed how Parkin is regulated by phospho‐Ub.^[31]^ The Ub−C3Br probe was also used to form a stable conjugate with Parkin, revealing a previously unidentified α‐helical region near the Rcat domain that guides the C‐terminus of Ub toward the Parkin catalytic site during E2‐E3 transthiolation.^[32]^ Ub‐Prg was used in reaction with the HECT‐type E3_HUWE1_ to obtain a structural mimic of an E3_HUWE1_~Ub complex, revealing the conformation of the C‐terminal tail of the ligase for the transfer of ubiquitin to an acceptor protein.^[23]^

### 3.1 Semisynthetic Ub Thioesters

To develop a tool to study E3 ligase catalytic activity while bypassing the need for E1 and E2 enzymes for Ub activation, Park and coworkers developed Ub~MESNa and the “Bypassing System”.^[33]^ While the previously described Ub‐derived probes act as suicide substrates, irreversibly trapping E3 and Ub, Ub~MESNa reacts directly with transthiolating E3 ligases to generate a catalytically active E3~Ub thioester. By reacting Ub~MESNa with a thiol‐equipped fluorescein molecule, this group produced the Ub~Fluor probe.[^34^, ^35^, ^36^] Like Ub~MESNa, this probe reacts with E3 to generate active E3~Ub. Additionally, the reaction liberates a free molecule of Fluorescein‐SH, providing a convenient means to measure E3~Ub production. It is important to note that while these probes can react with a wide array of transthiolating E3 ligases, they do not fully recapitulate the physiological complex for E3 activation: E2~Ub. Park et al. found that Ub transthiolation to a model HECT E3 was completed within seconds from E2~Ub, while Ub~MESNa had not completed transfer at 90 minutes, illustrating the importance of E2 for efficient E3~Ub formation.

### 3.2 “Cascading” Ub/Ubl Probe

Mulder and coworkers generated a unique “cascading” Ub‐ABP by substituting an electrophilic dehydroalanine (Dha) for glycine 76.^[37]^ After E1‐dependent adenylation, Ub‐Dha can be shuttled down the Ub cascade via thioester formation with E1 followed by transthiolation to E2 and subsequently to E3. However, at each step, it has the potential to react with individual active site cysteines via its Dha to produce trapped thioether‐linked Ub‐enzyme intermediates (Figure 2f). Structural studies of a thioether‐linked mimic of E2_UBE2D3_~Ub demonstrated that it was highly similar to a stable oxyester‐linked E2~Ub mimic (in which cysteine was mutated to serine). Importantly, this ABP has shown utility in labeling in live cells and mass‐spectrometry‐based proteome‐wide profiling. This latter approach revealed retrieval of E1s, nearly half of known E2s and two HECT‐type E3s from HeLa cell lysates. The coverage for E3s was somewhat limited, perhaps due to partial conversion to unreactive thioether at each transthiolation step or because most E3s exhibit higher specificities for native E2~Ub thioesters. The authors also demonstrated that the probe can be adapted to other Ubls by producing a NEDD8‐Dha. Subsequently, this group and others have expanded this strategy to many more Ubls, including SUMO, ISG15 and UFM1.[^38^, ^39^, ^40^] Mulder et al. report two methods for Ub‐Dha production: one from synthetic Ub^(1–75)^ produced by solid‐phase peptide synthesis, and the other from Ub^G76C^ (either synthetic or recombinant) reacted with 2,5‐dibromohexanediamide (45 % yield).^[37]^ More recently Qiao et al. reported a method to generate Ub‐Dha from recombinantly expressed Ub^G76C^, using 2‐nitro‐5‐thiocyanatobenzoic acid, resulting in Ub‐Dha at nearly 98 % yield after overnight reaction.^[39]^ This technique was also shown to be effective for other Ubls, provided that all native cysteines are substituted with other side chains to remove the thiol moiety. Overall, the ease of production methods and diverse array of applications make Ub‐Dha a powerful and versatile ABP for investigation of the ubiquitylation machinery.

The Ub/Ubl‐derivatives described thus far laid the groundwork for the development of other specialized probes with similar types of reactive groups and methods of synthesis, which are described in the following sections.

## 4 Tools to Study Ub/Ubl Activation

**Figure 3:**
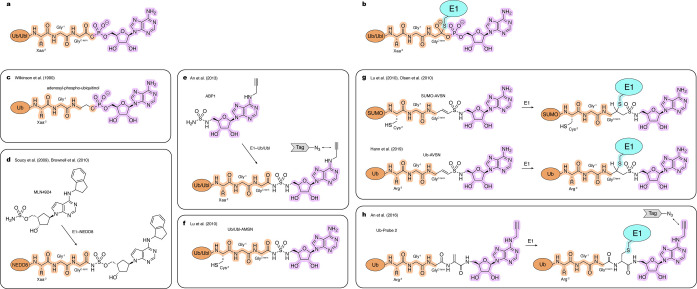
Tools to study Ub/Ubl activation by E1 enzymes. Atoms and linkages corresponding to native atoms of the last three residues at the Ub/Ubl C‐terminus, active site cysteine of an E1 enzyme and AMP are highlighted in orange, blue and pink, respectively. a, Native Ub/Ubl‐AMP complex. b, Native transition state of thiolation reaction. c–h, Examples of the chemical tools and strategies, as described in the text.

As mentioned earlier, Ub and Ubl proteins share similar biochemical activation mechanisms by E1 enzymes. To activate Ub, E1_UBA1_ first binds ATP^⋅^Mg^2+^ and Ub in the adenylation site and facilitates condensation between the C‐terminal carboxyl group of Ub and ATP. This reaction generates highly reactive Ub‐AMP, with the release of inorganic pyrophosphate (PP_*i*_^⋅^Mg^2+^) (Figure 3a). Subsequently, E1_UBA1_ undergoes extensive conformational remodeling around the Ub‐AMP for the nucleophilic attack by its catalytic cysteine (Figure 3b), to generate E1_UBA1_~Ub with the release of AMP from the adenylation site. Once formed, E1_UBA1_~Ub can bind a new set of substrates in the adenylation site to form a doubly Ub‐loaded complex. It has been shown that occupancy of the adenylation site with ATP^⋅^Mg^2+^ or Ub‐AMP is important for efficient transfer of thioester‐linked Ub to E1. Although consecutive steps of the E1_UBA1_ catalytic cycle were elucidated in a series of seminal biochemical studies,[^41^, ^42^, ^43^] the structural basis for this process remained unclear due to the transient nature of key intermediates.

### 4.1 Inhibitors of E1 Activation

Several non‐hydrolysable nucleotide analogues have been developed as inhibitors of Ub/Ubl E1s and as a strategy to gain functional insights and to obtain structural snapshots along the catalytic cycle. Wilkinson and coworkers developed a non‐hydrolyzable Ub‐AMP analogue, adenosyl‐phospho‐ubiquitinol (APU) with nanomolar binding competitive with ATP, that inhibits E1_UBA1_ activation in vitro (Figure 3c).^[44]^ Notably, this work also demonstrated a feasible method for coupling C‐terminal fragments to Ub through an aminolysis reaction with a corresponding C‐terminal acyl azide derivative of Ub. Similar semisynthetic strategies were later employed by others to generate other Ub‐derived probes.[^45^, ^46^] Although Ub/Ubl‐derived inhibitors like APU can be used in vitro, their usage to target E1 enzymes inside cells is limited due to their large molecular weight.

Cell permeable E1 inhibitors offer potential therapeutic benefit and are highly advantageous for studying Ub/Ubl cascades in a cellular context. One such compound, PYR‐41, displays micromolar potency against E1_UBA1_ in cells as reported by Yang and coworkers.^[47]^ Soucy and coworkers discovered MLN4924 (pevonedistat), an inhibitor with nanomolar cell potency against the NEDD8 E1 enzyme, using a combination of high‐throughput screening and medicinal chemistry.^[48]^ This discovery was complemented with a mechanistic study by Brownell and coworkers, showing that MLN4924 binds to the adenylation site and reacts with the E1_APPBP1/UBA3_~NEDD8 thioester forming an inhibitory adduct mimicking NEDD8‐AMP (Figure 3d).^[49]^

Using a similar strategy, other specific inhibitors of the SUMO E1 enzyme (ML‐792)^[50]^ and Ub E1_UBA1_ enzyme (TAK‐243/MLN7243) were developed.[^51^, ^52^] Furthermore, a pan‐specific E1 inhibitor from the same class, known as ′Compound 1′ and ABP A3, a dual inhibitor of the Ub and NEDD8 E1s, have also been reported.[^49^, ^53^] Leveraging reactivity for this class of compounds, An and coworkers developed activity‐based probes including ABP1 that targets a broad range of Ub/Ubl E1s and similarly forms covalent adducts with corresponding Ub/Ubl's in situ by virtue of an alkyne tag that can be conjugated to a fluorophore or affinity tag via click chemistry for detection and quantification (Figure 3e).^[54]^

### 4.2 Tools Used for Structural Studies of E1 Activation

To facilitate capture of key intermediates during E1 activation, Lu et al. developed Ub/Ubl probes containing C‐terminal 5′‐(aminodeoxy)adenosine‐5′‐N‐monosulfamide (AMSN) and 5′‐(aminodeoxy)adenosine‐5′‐N‐vinyl sulfonamide (AVSN) (Figure 3f,g).^[55]^ While both Ub/Ubl‐AMSN and Ub/Ubl‐AVSN are non‐hydrolysable mimics of the Ub/Ubl adenylate, Ub/Ubl‐AVSN contains an additional vinyl group positioned to form a covalent adduct with the catalytic cysteine of the E1 that mimics the transition state formed during thioester bond formation (Figure 3b,g). The utility of these probes was demonstrated in studies by Olsen and coworkers, where SUMO‐AMSN and SUMO‐AVSN were used to obtain structures of the E1_SAE1/UBA2_ that illuminated conformational changes accompanying SUMO activation and thioester bond formation.^[56]^ In a subsequent study, Hann et al. used analogous Ub‐derived probes to obtain and study corresponding complexes of the Ub E1_Uba1_ enzyme.^[45]^ This study introduced an alternative acyl azide‐mediated method for synthesizing Ub‐AVSN compared to the native chemical ligation method used to generate SUMO‐AMSN and SUMO‐AVSN. This alternative method facilitates the installation of AVSN with high yield and eliminates the need to introduce cysteine at the ligation site, preserving the native Arg residue of Ub (Arg^−2^) (Figure 3g).

An alternative strategy to capture intermediates during thioester bond formation entailed development of a probe that employed dehydroalanine chemistry as described by An and coworkers.^[57]^ In this approach, a non‐hydrolyzable AMP analogue was first installed at the C‐terminus of a Ub/Ubl through native chemical ligation which leaves a cysteine residue at the ligation site. This cysteine was then converted to dehydroalanine using 2,5‐dibromohexanediamide. The dehydroalanine can react with the active site cysteine of E1. This strategy was used with Ub and LC3 and their corresponding E1s (UBA1 and ATG7) (Figure 3h). While this approach yields a product that is less isosteric relative to the native intermediate or AVSN‐based probes, a major benefit is the introduction of an azide group that allows for labeling and detection of the trapped intermediate.

## 5 Enzyme~Ub/Ubl Thioester Mimics

**Figure 4:**
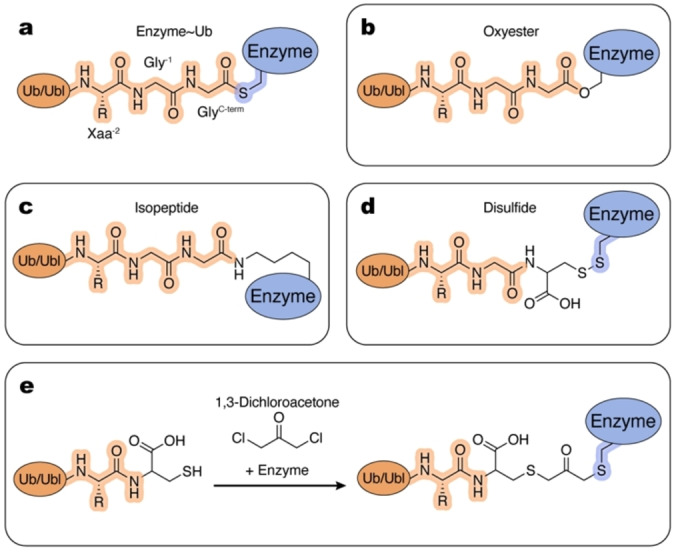
Tools and methods to mimic Enzyme~Ub/Ubl intermediates. Atoms and linkages corresponding to native atoms of last three residues at the C‐terminus of Ub/Ubl and active site cysteine of an enzyme are highlighted in orange and blue, respectively. a, Native Enzyme~Ub/Ubl intermediate. b–e, Examples of the chemical tools and strategies, as described in the text.

The enzymatic cascade through which a Ub/Ubl is conjugated to a substrate protein involves Ub/Ubl~enzyme intermediates, wherein the Ub/Ubl C‐terminal carboxylate is tethered to the enzyme's active site cysteine via a thioester bond (Figure 4a). The high‐energy, labile nature of this bond is an important biochemical feature of these conjugation intermediates, enabling rapid, unidirectional transfer through the cascade from E1 to substrate. Unfortunately, the thioester bond is prone to hydrolysis which makes these intermediates difficult to capture and study. To overcome this difficulty, researchers developed a variety of chemical substitutes to produce a more stable linkage between the Ub/Ubl and enzyme, facilitating detailed biochemical and structural characterization of these conjugation intermediates and their myriad binding and reaction partners. These thioester mimetics vary in terms of chemical stability, route of synthesis, and degree of similarity to native thioester linkages.

Some E2~Ub/Ubl mimics can be produced by single amino acid substitutions. One of the closest chemical mimics of the native thioester is the oxyester (Figure 4b). Sung et al. and Sullivan et al. first showed that E2 enzymes with active site cysteine to serine substitution can form an oxyester bond to Ub when reacted with E1~Ub under standard conditions.[^58^, ^59^] While the oxyester is formed at a slower rate than the thioester, it is also more stable. Miura et al. used E2_UBE2B_ (C88S) to generate the oxyester mimic for the first structural study of an E2~Ub conjugate.^[60]^ Subsequently, many groups have employed this approach for structural and biochemical studies of E2~Ub/Ubls, both in isolation and in complex with interaction partners such as E3s, substrates, and other proteins.[^61^, ^62^, ^63^, ^64^, ^65^, ^66^, ^67^, ^68^, ^69^, ^70^, ^71^, ^72^, ^73^, ^74^, ^75^, ^76^, ^77^, ^78^, ^79^, ^80^, ^81^, ^82^, ^83^, ^84^, ^85^, ^86^, ^87^]

While the oxyester linkage is more stable than the native thioester, it is still susceptible to hydrolysis, particularly in the presence of RING E3s that activate the E2~Ub/Ubl.^[88]^ To overcome this obstacle and determine the structure of a RING E3 bound to E2~Ub, Plechanovová and coworkers substituted the E2_UBE2D1_ catalytic cysteine to lysine, which, upon transfer from E1~Ub at high pH, generates a stable isopeptide linkage between the Ub C‐terminus and E2 active site (Figure 4c). This E2~Ub mimic was used to obtain a structure in complex with E3_RNF4_ providing important insights to activation of E2~Ub by RING E3s.^[88]^ Despite the increased length of the lysine side chain relative to cysteine, this strategy successfully captured the activated conformation of E2‐Ub, one that closely matches E2‐Ub conformation and active site configuration observed in the structure of the oxyester mimic of E2_UBE2D2_~Ub in complex with E3_BIRC7_.[^67^, ^88^] The ease of implementing this approach facilitated numerous structural studies of other E2~Ubs/Ubls in complex with E3s and other binding partners.[^30^, ^71^, ^74^, ^75^, ^78^, ^88^, ^89^, ^90^, ^91^, ^92^, ^93^, ^94^, ^95^, ^96^, ^97^, ^98^, ^99^, ^100^, ^101^, ^102^, ^103^, ^104^, ^105^, ^106^, ^107^, ^108^, ^109^, ^110^, ^111^, ^112^, ^113^, ^114^, ^115^, ^116^, ^117^, ^118^, ^119^, ^120^, ^121^, ^122^, ^123^, ^124^]

Oxyester and isopeptide linkages are produced via a single amino acid substitution of the E2 catalytic cysteine, but substitutions to the Ub/Ubl moiety can also be used to make stable thioester mimetics of the E2~Ub/Ubl. Substitution of the Ub/Ubl C‐terminal glycine to cysteine can be used to produce a disulfide bond between E2 and Ub/Ubl (Figure 4d). By combining reduced Ub^G76C^ and reduced yeast E2_UBC1_ under oxidizing conditions with Cu^2+^, Merkley et al. produced a stable E2~Ub thioester mimic to study by NMR spectroscopy.^[125]^ This approach may require substitution of E2 cysteines other than the active site cysteine to prevent undesired disulfide bond formation. Cysteine to serine substitutions of the two non‐catalytic cysteine residues in E2_UBE2L6_ allowed Serniwka and Shaw to determine the structure of disulfide‐linked E2_UBE2L6_~Ub by NMR.^[126]^ This approach facilitated study of E2~Ub thioester mimics and their intermolecular interactions with E3s and other proteins.[^127^, ^128^, ^129^, ^130^, ^131^, ^132^] Adapting a method originally used to produce linkage‐specific diubiquitin molecules, Wiener et al. generated an E2_UBC13_~Ub conjugate that is stable in reducing conditions, by reacting Ub^G76C^ and E2 with dichloroacetone (Figure 4e).[^66^, ^133^] This E2~Ub thioester mimic was crystallized with the deubiquitinase OTUB1 to enable structural and biochemical characterization.

Finally, certain thiol‐reactive Ub/Ubl‐derived ABPs have been used to obtain mimics of thioesters (Figure 2b–f). These studies are described in section 3 of this review, “Thiol‐Reactive Ub/Ubl‐Derived ABPs”.

Several parameters must be considered when deciding between approaches for E2~Ub/Ubl thioester mimetics, including stability in the buffer(s) of choice, molecular similarity to the native linkage, and ease of production. In their NMR studies, Choi et al. found that a disulfide‐linked E2_UBE2R1_~Ub failed to recapitulate the contacts between E2 and Ub that were observed with the equivalent oxyester‐linked species.^[64]^ Therefore, in addition to weighing the various pros and cons of these strategies, it may be necessary to empirically determine if the thioester mimic is a true analogue of any given enzyme~Ub/Ubl adduct.

## 6 Tools and Strategies to Study Transthiolation Intermediates

Transthiolation reactions are used to transfer thioester‐activated Ub/Ubl from E1 to E2 enzymes, and from specific E2s to transthiolating E3s. Notably, in a unique mechanism observed in the E3 ligase MYCBP2, the founding member of the RING‐Cys‐Relay (RCR) family of E3 ligases, transthiolation is used to transfer thioester‐activated Ub between two cysteines within a single enzyme.[^9^, ^134^]

It is perhaps unsurprising that steps involving transthiolation may also serve as points of regulation in specific pathways. For instance, certain RBR and HECT E3s possess autoinhibitory domains which can mask the active site, inhibiting E2‐E3 transthiolation.[^24^, ^28^, ^29^, ^30^, ^31^, ^135^, ^136^, ^137^, ^138^, ^139^, ^140^] Another mode of regulation of transthiolating E3 activity involves binding of adaptor proteins that recruit charged E2 enzymes to HECT E3s, enhancing E2‐E3 transthiolation.^[141]^

**Figure 5:**
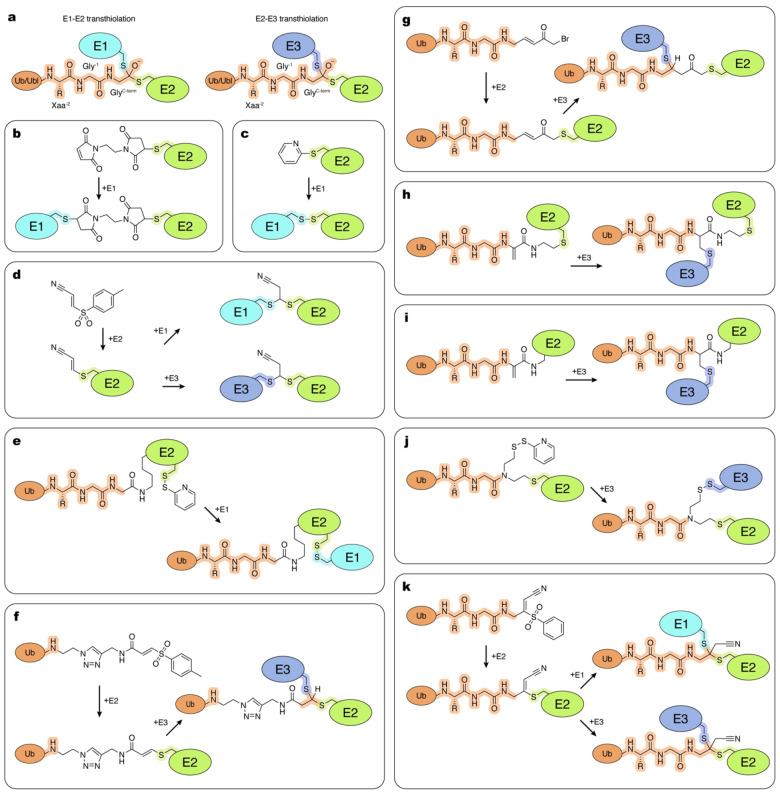
Tools and methods to trap transthiolation. Atoms and linkages corresponding to native atoms of last three residues at the C‐terminus of Ub/Ubl, active site cysteine of an E1 enzyme, active site cysteine of an E2 enzyme and active site cysteine of an E3 enzyme are highlighted in orange, cyan, green and blue, respectively. a, Transition states formed during transfer of Ub/Ubl from E1 to E2 (left), and transfer of Ub from E2 to transthiolating E3 (right). b–k, Examples of the chemical tools and strategies, as described in the text.

Transthiolation reactions are presumed to proceed through formation of tetrahedral transition states which link the active site cysteine sulfur atoms of two enzymes via the C‐terminal carbonyl carbon atom of Ub/Ubl (Figure 5a). Native complexes between enzymes containing thioester‐linked Ub/Ubl and their respective thioester accepting enzymes are unstable because Ub/Ubl is readily passed from the catalytic cysteine of one enzyme to another. Due to the short lifetimes and lability of transthiolation intermediates, such complexes are difficult to characterize as are the molecular details underpinning directionality in the transthiolation reactions.

### 6.1 Catalytically Inactive Enzymes

Structural studies aimed at capturing intermediates during transthiolation have employed catalytically inactive enzymes and/or stable mimics of donor Ub/Ubl~enzyme complexes (discussed in section 5, “Enzyme~Ub/Ubl Thioester Mimics”). Huang and coworkers used a native E1_APPBP1/UBA3_~NEDD8 thioester and a catalytically inactive E2 harboring a cysteine‐to‐alanine substitution that prevents transfer of NEDD8 to obtain a structure of a complex that captured interactions required for transthiolation.^[142]^ While this structure revealed important aspects of E1 and E2 interactions during transthiolation, the reaction mechanism remained unclear as the active sites were separated by more than 20 Å.^[142]^ Kamadurai and coworkers used an oxyester mimic of E2~Ub in complex with the HECT‐type ligase E3_NEDD4_ to obtain a structure that captured interactions required for transthiolation.^[62]^ In this case, the active site cysteine in the E3 was substituted with serine or alanine to prevent transfer of oxyester‐linked Ub, and amino acid substitutions in E2 were introduced to stabilize the complex with E3. This landmark structure revealed important determinants for E2‐E3 transthiolation, including crucial structural rearrangements in E3 that bring the two active sites together to facilitate transfer of Ub as well as determinants on the E3 that coordinate Ub after transfer. While this structure captures numerous hallmark features of E2‐E3 transthiolation, atoms corresponding to the active site cysteine sulfurs are separated by ~8 Å, a distance longer than that required for transthiolation.^[62]^ Lechtenberg and coworkers provided insight into an E2‐RBR‐type E3 transthiolation complex by using E2_UBE2D2_ with its catalytic cysteine substituted to lysine and conjugated to Ub to form a stable complex between the E2_UBE2D2_~Ub mimic and E3_RNF31_.^[96]^ While the carbonyl carbon atom of Ub's G76 is displaced by ~4 Å due to the cysteine 85 to lysine substitution in the E2_UBE2D2_~Ub mimic, atoms relevant to active site sulfurs are separated by ~5 Å, just 2–3 Å longer than the estimated distance spanned by the tetrahedral intermediate.

### 6.2 Crosslinking of Active Site Cysteines

Crosslinking active site cysteines represents another major approach to generate mimics of transthiolation complexes. Kaiser and coworkers used the homobifunctional crosslinker bismaleimidoethane to crosslink active site cysteines and obtain structures of the E1_Atg7_–E2_Atg3_ and E1_Atg7_–E2_Atg10_ complexes.^[143]^ While this approach can stabilize interactions for structural and functional studies, the crosslinker introduces a spacer between reactive centers containing two bulky maleimide rings (Figure 5b).

In another example, 2,2′‐dipyridyldisulfide was used to derivatize the thiol active site cysteine of E2_Ubc4_ to induce formation of a disulfide with the active site cysteine of E1_Uba1_. This strategy brings the active sites into proximity at a distance that is one bond shorter than the native tetrahedral intermediate (Figure 5c).^[144]^ The linkage is redox‐sensitive but stable enough for structural studies, and was used to provide structural information on underlying E1–E2 interactions. This strategy was subsequently used by Lv and coworkers to obtain a structure of E1_Uba1_–E2_Ubc15_,^[145]^ and by Williams and coworkers to obtain a structure of E1_Uba1_–E2_Cdc34_.^[107]^ More recently, Wallace and coworkers utilized this disulfide trapping strategy to obtain structures for E1_UBE1L_–E2_UBE2L6_ in the ISG15 pathway.^[146]^

To develop a redox‐stable crosslinking strategy that could recapitulate the same distance between active site cysteines as in native intermediates, Stanley and coworkers investigated the reactivity of biselectrophilic tosyl‐substituted doubly activated enes (TDAEs).^[22]^ One of the compounds studied was 3‐tosylacrylonitrile (BAY 11–7082). Originally discovered as a putative kinase inhibitor,^[147]^ BAY 11–7082 was later shown to form inhibitory covalent acrylonitrile adducts with cysteines of human E2_Ubc13_ and E2_Ubc7_ and the RBR‐type E3 ligase HOIP.^[148]^ A subsequent study demonstrated regiospecific bis‐addition of thiols to related 3‐(phenylsulfonyl) acrylonitrile.^[149]^ Leveraging these properties, Stanley and coworkers demonstrated that reaction of BAY 11–7082 with E2_UBE2N_ resulted in formation of an E2_UBE2N_‐acrylonitrile adduct that can be crosslinked to E1_UBA1_ to form a dithioacetal complex. While lacking the Ub/Ubl molecule itself, this crosslinking strategy recapitulates the native distance between active centers and tetrahedral geometry of the transient transthiolation intermediate (Figure 5d). This work paved the way for the development of probes that capture tetrahedral mimics of the intermediates during transthioesterification.[^150^, ^151^]

### 6.3 Strategies that Link all Reaction Components

Research groups continue to focus on developing strategies to capture mimics of transthiolation complexes that include all protein components isosterically linked within the reaction center to mimic native transition states. These strategies usually rely on two general design principles. The first involves disulfide‐linking the two active sites while introducing a Ub/Ubl molecule to a lysine installed proximal to the active site of E2 (Figure 5e). This strategy, first reported by Streich and Lima to capture a substrate/RING E3/E2~SUMO complex (discussed in section 7, “Trapping E2~Ub/Ubl:Substrate and Ub~E3:Substrate Complexes”), was employed by Yuan et al. to capture a structure of an E1_Uba1_~Ub~E2_Cdc34_ mimic.[^152^, ^153^] More recently, Afsar and coworkers applied the analogous strategy to capture a complex between E1_UBE1L_ and E2_UBE2L6_ in the ISG15 conjugation cascade.^[154]^ Unexpectedly, the non‐native linkage resulted in a complex with the ISG15 moiety of the E2~ISG15 mimic positioned on the opposite side of the E1 complex relative to Ub in an E1_Uba1_~Ub~E2_Cdc34_ mimic. Given the high degree of structural similarities between the Ub and the ISG15 systems, it remains unclear if this is an artifact of the crosslinking strategy employed. Further structural studies using strategies that accurately mimic transthiolation intermediates will be required to confirm or refute these models.

A second major approach to capturing complexes closer to the native transition state involves strategies that covalently combine active site thiols and the C‐terminal carbonyl carbon atom of the respective Ub/Ubl (Figure 5a). Several E2‐Ub ABPs and chemical strategies have been developed with this goal in mind. By including E2 as part of their recognition element, E2‐Ub ABPs reduce reactivity with DUBs, mimic relevant substrates for E3 ligases, and were shown to react more efficiently with E3s relative to isolated Ub‐derived probes or E2‐ABPs.[^21^, ^150^] An E2‐Ub ABP developed by Pao and coworkers harbors an activated vinylsulfide (AVS) electrophile that reacts with E3 catalytic cysteines, forming a stable transthiolation mimetic.^[150]^ Copper‐catalyzed azide‐alkyne cycloaddition was used to generate a Ub^(1–73)^–tosyl‐substituted doubly activated ene (Ub‐TDAE) with E2 to form E2‐Ub‐AVS. Pao and coworkers demonstrated that this ABP reacted specifically with E3 ligases and used it to better understand the determinants for transthiolation to E3_Parkin_ in vitro and in cellular extracts. This ABP is conveniently modular: once the Ub‐TDAE is generated, any E2 (providing that other non‐active site cysteines are substituted) can be reacted under native conditions to form an E2‐Ub‐AVS adduct. Subsequent reaction with a respective E3 captures a stable mimic at a native distance between the thiols of the active site cysteines. While powerful, this method replaces the last three residues and side chains of the Ub C‐terminus with a linker containing triazole (Figure 5f). In the subsequent study, Pao and coworkers introduced an alternative version of the Ub‐TDAE probe built using Ub^(1–74)^. In this variant, the Ub portion is extended to include the native Arg74 residue, but the synthetic portion of the triazole linkage remains unchanged, resulting in a spacer that is five atoms longer compared to the native linkage with Ub.^[9]^ Importantly, E2‐Ub‐AVSs obtained by reaction of the Ub‐TDAE probes with E2s were used for activity based profiling in cell extracts. Most notably, this led to the discovery of MYCBP2, a defining member of the new RING‐Cys‐Relay (RCR) class of E3 ligases, that utilizes unprecedented intramolecular transthiolation of Ub to promote Ub modification of hydroxyl groups.^[9]^ In a follow‐up study, Mabbit and coworkers employed Ub‐TDAE to determine a structure with MYCBP2 which recapitulated the native geometry of the transthiolation tetrahedral intermediate for the first time.^[134]^

Horn‐Ghetko and coworkers used an intein‐based semisynthetic method to generate an ABP with a Michael acceptor between the C‐terminus of Ub and the active site of E2_UBE2L3_.^[26]^ In this approach, Ub~MESNa is reacted with (E)‐3‐[2‐(bromomethyl)‐1,3‐dioxolan‐2‐yl]prop‐2‐en‐1‐amine (BmDPA), which upon subsequent deprotection yields a reactive α‐bromo‐vinylketone group.^[155]^ This reactive Ub is subsequently reacted with active site cysteine in E2 (providing that other non‐active site cysteines are substituted). Importantly, this strategy maintains the C‐terminal residues of Ub and the transthiolation mimetic produced upon reaction with RBR E3_ARIH1_ positions the E3 active site cysteine only three atoms further from the native linkage (Figure 5g). This strategy allowed the capture and structural determination of the E2‐E3 transthiolation complex with E3_ARIH1_ when assembled with a neddylated cullin‐RING E3 ligase, providing important insights to its catalytic cycle.^[26]^

To expand the E2‐Ub ABP toolkit, Xu et al. used native chemical ligation and sequential Dha formation to produce an E2‐Ub‐Dha ABP by combining Ub generated by solid phase peptide synthesis and recombinantly expressed E2.^[156]^ This ABP maintains Ub's native C‐terminus and effectively labels HECT E3s, but requires refolding after synthesis. The ternary complex resulting from this probe positions E3 Cys and Ub C‐terminus by an equivalent number of atoms as in the native intermediate, while the E2 Cys is positioned four and five atoms further away from the Ub C‐terminus and E3 Cys, respectively (Figure 5h). Subsequently, a similar ABP was produced by Liang et al. through chemical synthesis of E2 with a 2,3‐diaminopropionic acid (Dap) unit installed at the active site cysteine, which was used in a reaction with Ub^(1–75)^~MESNa to position the Dha warhead at Ub Gly 76.^[157]^ Reacting this probe with E3 yields a covalent complex that more closely mimics the native transthiolation intermediate, bringing the E2 closer to the Ub and E3, as compared to the product of the probe developed in Xu et al. (Figure 5i). Recently, Zheng et al. generated an ABP using a bifunctional CAET molecule.^[158]^ Using recombinant E2 and Ub^(1–75)^~MESNa, this ABP can form a disulfide bond with E3 Cys.^[159]^ In the resulting transthiolation mimetic, the E2 and Ub are at the correct distance relative to each other, but the E2 and E3 sulfurs are separated by five additional atoms relative to the native complex (Figure 5j). Additionally, this strategy is limited by the need to form the E2‐Ub probe under denaturing conditions, restricting the repertoire of E2s to those that can be refolded in vitro. Finally, building upon previous work by Stanley et al. Delos Reyes and coworkers developed the bis‐electrophilic Ub‐PSAN (3‐[phenylsulfonyl]‐4‐aminobut‐2‐enenitrile).[^22^, ^46^] This probe can be reacted with E2 to yield E2‐Ub‐AVS in a similar fashion as the Ub‐TDAE probe.^[150]^ The resulting E2‐Ub‐AVS can be then reacted with other enzymes to yield atomically tailored dithioacetal analogues of transthiolation intermediates with substitution of the Ub C‐terminal Gly76 oxyanion by a cyanomethyl group (Figure 5k).[^46^, ^151^] Using this strategy, Kochańczyk and coworkers determined a continuum of cryo‐EM structures for mimics of E1‐E2 and E2‐E3^HECT^ transthiolation intermediates to reveal new mechanistic insights and conformational transitions underlying Ub transfer in the E1–E2 and E2–E3^HECT^ transthiolation reactions.^[151]^

## 7 Trapping E2~Ub/Ubl:Substrate and Ub~E3:Substrate Complexes

**Figure 6:**
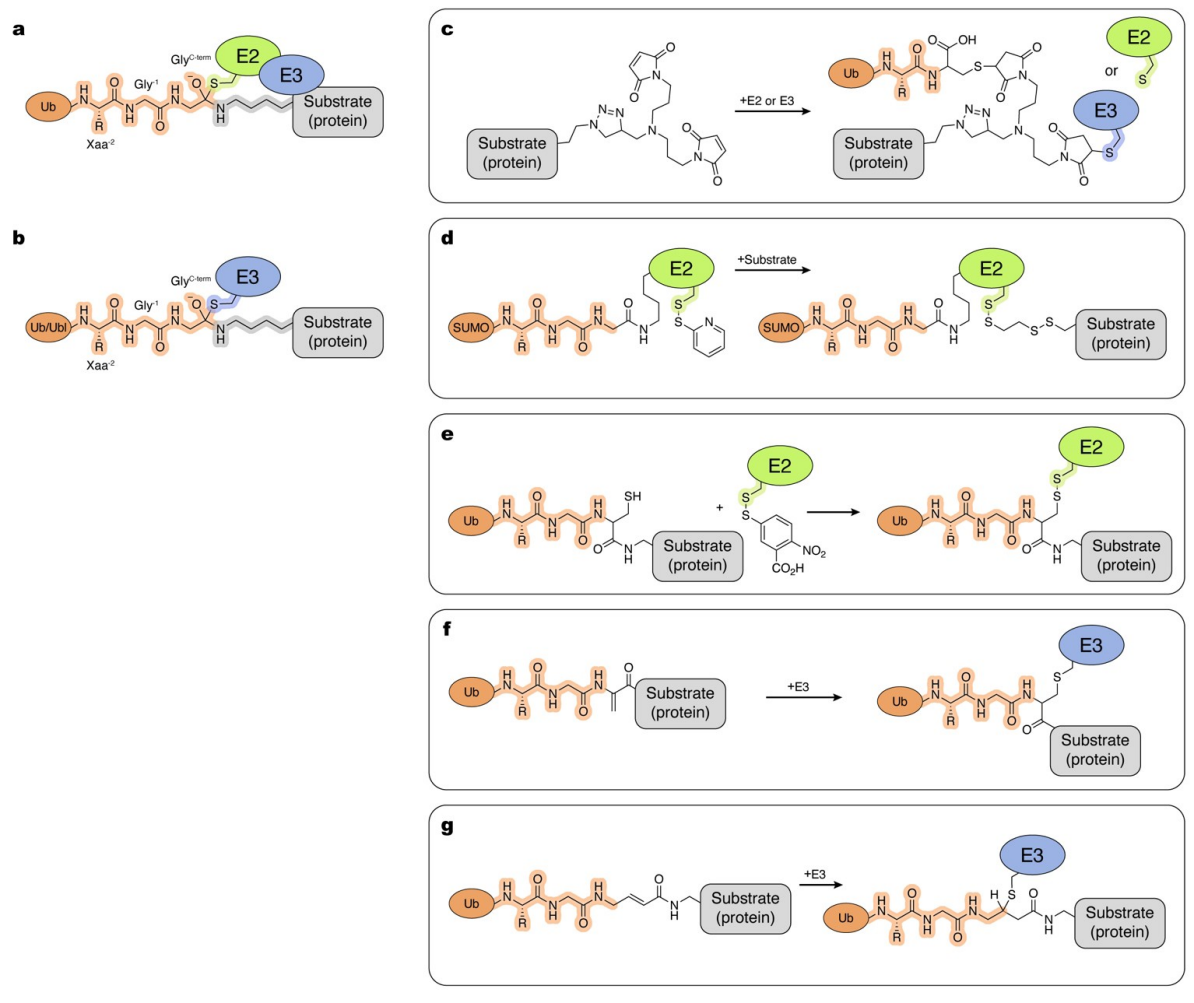
Tools and methods to trap Ub/Ubl conjugation. Atoms and linkages corresponding to native atoms of last three residues at the C‐terminus of Ub/Ubl, active site cysteine of an E1 enzyme, active site cysteine of an E2 enzyme and active site cysteine of an E3 enzyme are highlighted in orange, cyan, green and blue, respectively. a, Tetrahedral intermediate formed during Ub/Ubl conjugation by scaffold E3s. b, Tetrahedral intermediate formed during Ub/Ubl conjugation by transthiolating E3s. c–g, Examples of the chemical tools and strategies, as described in the text.

The final step in Ub/Ubl conjugation entails transfer of the thioester bond between the Ub/Ubl C‐terminus and E2/E3 cysteine to link Ub/Ubl to its substrate, usually through formation of an isopeptide bond to a target lysine residue (Figure 6a,b). Here, we emphasize methods developed to mechanistically characterize Ub/Ubl conjugation at the structural level and to study E3 ligase activity. The rapidly growing number of non‐ABP‐based methods to identify E3 ligase substrates or E3 ligases targeting a specific substrate are beyond the scope of this review. We encourage readers new to this area to consult other review articles published recently.[^160^, ^161^]

Various chemical crosslinking strategies were developed to trap stable mimics of substrate modification reaction intermediates. A tripartite chemical crosslinking approach was employed by Kamadurai and coworkers to link an E2/E3 active site to the Ub/Ubl C‐terminus and substrate (Figure 6c).^[162]^ A peptide substrate was synthesized with the target lysine substituted by azidohomoalanine and linked via click chemistry to a homobifunctional bismaleimide reagent, which is crosslinked by one arm to an E2/E3 active site cysteine, and by the other arm to a C‐terminal cysteine of a Ub containing a Gly75Cys substitution. This technique enabled structure determination of a HECT E3 crosslinked with Ub and substrate as well as a crosslinked E2~Ub‐substrate for structural studies of a RING‐type E3 within the APC complex.[^162^, ^163^, ^164^] While useful in capturing protein‐protein interactions in these complexes, the bulky nature of this crosslinker likely perturbs molecular interactions within the active site as exemplified in the crystal structure for the HECT E3 complex which lacked electron density for the crosslinker and Ub C‐terminal residues. Nevertheless, this tripartite crosslinking method enabled capture of these normally transient complexes.

Streich and Lima devised an alternative strategy to stably crosslink an E2~SUMO:substrate complex. Generating an Ala129Lys substitution at the E2_Ubc9_ active site enabled the formation of an isopeptide‐linked mimic of E2~SUMO, while leaving the E2 active site cysteine available for crosslinking. With the substrate target lysine substituted to cysteine, a bifunctional, thiol reactive reagent can then be used to link the substrate and E2 (Figure 6d). This crosslinked subcomplex was then combined with the RING‐type E3_Siz1_ for structure determination of the complex.^[152]^ This strategy facilitates trapping of protein substrates produced via recombinant expression systems under native conditions. Moreover, use of ethanedithiol (EDT) generated a crosslinked product wherein the substrate and E2 active site are separated by the same number of bonds as in the native transition state. While this approach is relatively easy to implement, it does not mimic a bona fide tetrahedral intermediate and relies on accommodating an additional lysine side chain within the active site. The Ala129Lys E2~Ubl thioester mimetic strategy was subsequently used to determine a crystal structure of a complex with yeast E3_Nse2_ and was successfully adapted for the human E2_UBC9_ as well.[^165^, ^166^]

Advancements continue to emerge in the pursuit of tripartite complexes between E2, Ub/Ubl C‐terminus, and substrate. Baek and coworkers generated one such crosslinked species for structure determination of a Cullin‐RING ligase complex.^[167]^ First, Ub^(1–75)^~MESNa (described earlier in this review) is joined to a substrate peptide, harboring an N‐terminal cysteine in place of the target lysine, via native chemical ligation (NCL). E2 catalytic cysteine is activated with 5,5‐dithio‐bis‐(2‐nitrobenzoic acid) (DTNB/Ellman's reagent) and then reacted with the Ub‐substrate peptide to produce the final crosslinked product (Figure 6e). Following a similar procedure, but using 2,5‐dibromohexanediamide instead of DTNB, Horn‐Ghetko and coworkers converted the cysteine to dehydroalanine.^[26]^ Incubating this reactive Ub‐substrate with the RBR‐type E3 ARIH1 in complex with its Cullin‐RING ligase binding partners, produced a stably crosslinked complex for analysis by cryo‐EM, with E3 and substrate separated by only one additional atom as compared to the native intermediates (Figure 6f). In the same study, an alternative approach utilized a fully synthetic Ub substate probe. However the trapped complex was not used for structural studies (Figure 6g).

### 7.1 Photocrosslinking Probes

Transthiolating E3 ligases, such as those of the HECT and RBR families, can be captured by ABPs that trap the active E3 by reacting with a cysteine nucleophile. However, the majority of E3 ligases function as molecular scaffolds that stabilize the catalytically active conformation of the E2‐Ub thioester to promote transfer of Ub directly from E2 to substrate. Therefore, alternative designs are needed for ABPs that can trap activated scaffold E3s. To this end, researchers developed E2‐Ub probes with photoactivatable crosslinkers positioned to react with E3 ligases upon formation of the ternary complex.

Krist and Statsyuk produced a single cysteine substitution E2 or E2‐Ub oxyester mimetic, alkylated with a pH‐cleavable diazirine photocrosslinker.^[168]^ Upon UV irradiation and probe cleavage, they used mass spectrometry to identify E3 residues previously unknown to be involved in catalysis. Zhang et al. generated a photocrosslinking probe from an E2~SUMO mimic.^[169]^ This photoaffinity probe was produced from recombinant SUMO and a chemically synthesized SUMO E2 enzyme. The novel strategy combined three orthogonal peptide ligation methods to generate a final E2‐SUMO probe with an N‐terminal biotin for purification, a stable, amide‐linked SUMO at the active site with Dap substituted for Cys, and a diazirine photocrosslinker installed at one of several sites. With the diazirine at Ubc9 F22, the SUMO E3 ligase RanBP2 was successfully trapped upon UV irradiation.

Lastly, a photocrosslinking ABP was developed by Mathur et al. to capture scaffold‐type E3 ligases.^[170]^ This group recombinantly expressed ubiquitin with a photocrosslinking, unnatural p‐benzoyl‐L‐phenylalanine (Bpa) amino acid incorporated via an evolved tRNA synthetase‐tRNA pair, and modified the N‐terminus with a biotin affinity tag. This Ub was then activated by E1 and transferred to a Cys to Lys E2 mutant, generating a stable, isopeptide‐linked E2‐Ub ABP. The resulting probe was shown to react specifically with the activated forms of the RING E3 ligases RNF4 and c‐Cbl, and captured numerous other E3 ligases from cell extracts specifically in response to their activation stimuli, thereby demonstrating the hallmarks of a bona fide ABP. This probe was also shown to be effective at reporting relative activities of various pathogenic mutants of the RNF12 E3 ligase.^[171]^

In all, several groups devised chemical probes, both with recombinantly expressed and chemically synthesized proteins, to study scaffold‐type E3s. Utilizing various mimics of the E2‐Ub thioester equipped with an appropriately positioned photocrosslinker will continue to enable researchers to gain insights into structures and biochemical mechanisms of Ub and Ubl E3s to provide a platform by which to study E3 mutations or develop screens to discover molecules that could alter their activity.

## 8 Summary and Outlook

Chemical tools and methods discussed in this review have proven instrumental in uncovering mechanisms governing Ub and Ubl conjugation pathways. These tools and methods, ranging from activity‐based probes, inhibitors, and other approaches successfully capture mimics of fleeting intermediates, resulting in significant breakthroughs over recent years. We now possess an impressive chemical toolbox, but the discovery of new mechanisms operating in Ub/Ubl conjugation pathways necessitates further development of these tools.

One notable feature of Ub/Ubl conjugation pathways is the vast combinatorial and structural diversity of the transient enzymatic complexes formed. Chemical tools are most widely used in the structural characterization of stable mimics of these transient complexes. These studies rely on the principle that introducing mimics of the intermediates or transition states will capture complexes and active sites in configurations that reflect native states. While all strategies discussed, regardless of their degree of isostery, have contributed to our understanding of intermediates in the Ub/Ubl conjugation cascades, it is also evident that mechanistic predictions are limited by the degree of structural mimicry achieved by the respective analogue. This is corroborated by an elegant study by Liwocha and coworkers, demonstrating that various chain‐forming E2 and E3 enzymes are strikingly sensitive to even a single methylene difference in the length of the lysine hydrocarbon linker relative to the acceptor Ub.^[172]^ It is expected that similar specificities are required in other enzymatic reactions within Ub/Ubl conjugation cascades. Therefore, further development of methods for trapping complexes will continue to rely on developing tools that are as isosteric as possible relative to their native counterparts.

## Conflict of Interests

The authors declare no competing interests.

9

## Biographical Information

*Tomasz Kochańczyk is a postdoctoral researcher in the Dr. Christopher D. Lima lab at the Sloan Kettering Institute in New York City, studying structural and biochemical mechanisms of post‐translational modification by ubiquitin and ubiquitin‐like proteins. He received his PhD in biochemistry from the University of Wroclaw under the supervision of Dr. Artur Krężel for his structure‐function studies of the zinc hook domain of the Rad50 protein. During his PhD, he spent six months at the Sloan Kettering Institute, where he worked with Dr. John H. Petrini on the roles of Rad50 in the DNA damage response*.

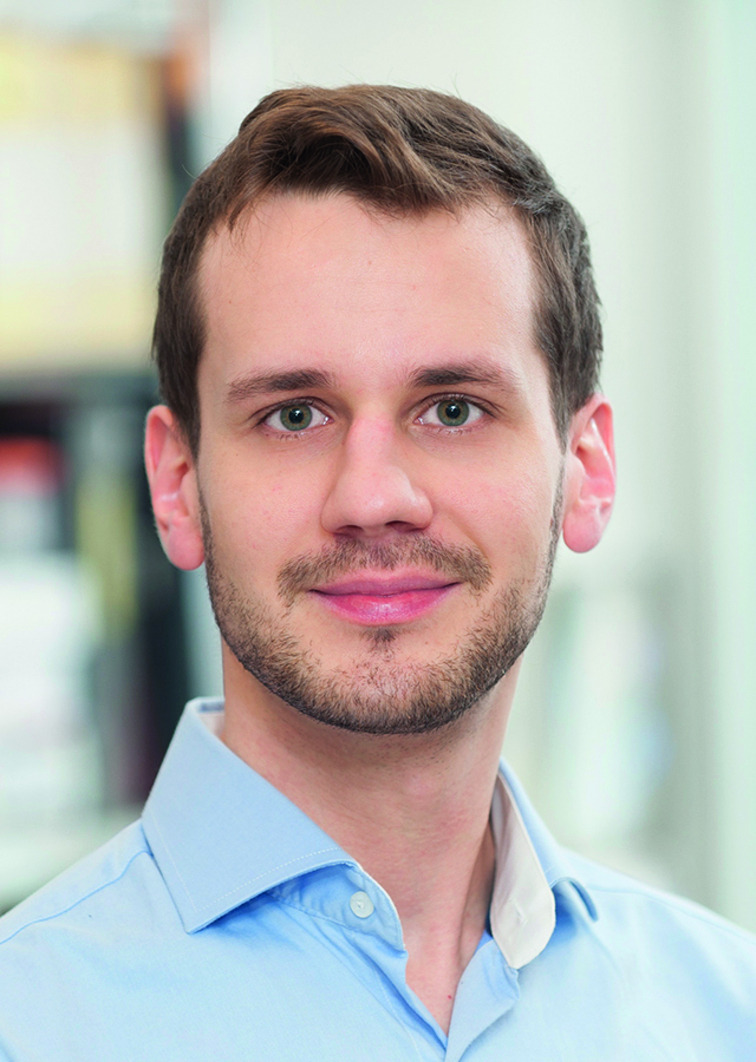

## Biographical Information

*Michael Fishman graduated from the University of Southern California with a Bachelor of Arts in Neuroscience. Subsequently, he worked as a postbaccalaureate student in the lab of Dr. Lloyd Fricker at the Albert Einstein College of Medicine in the Bronx, New York. He recently completed his PhD working under the supervision of Dr. Christopher D. Lima at the Sloan Kettering Institute, reconstituting the SUMO modification of DNA Topoisomerase I by PIAS4 to uncover molecular determinants of the reaction*.

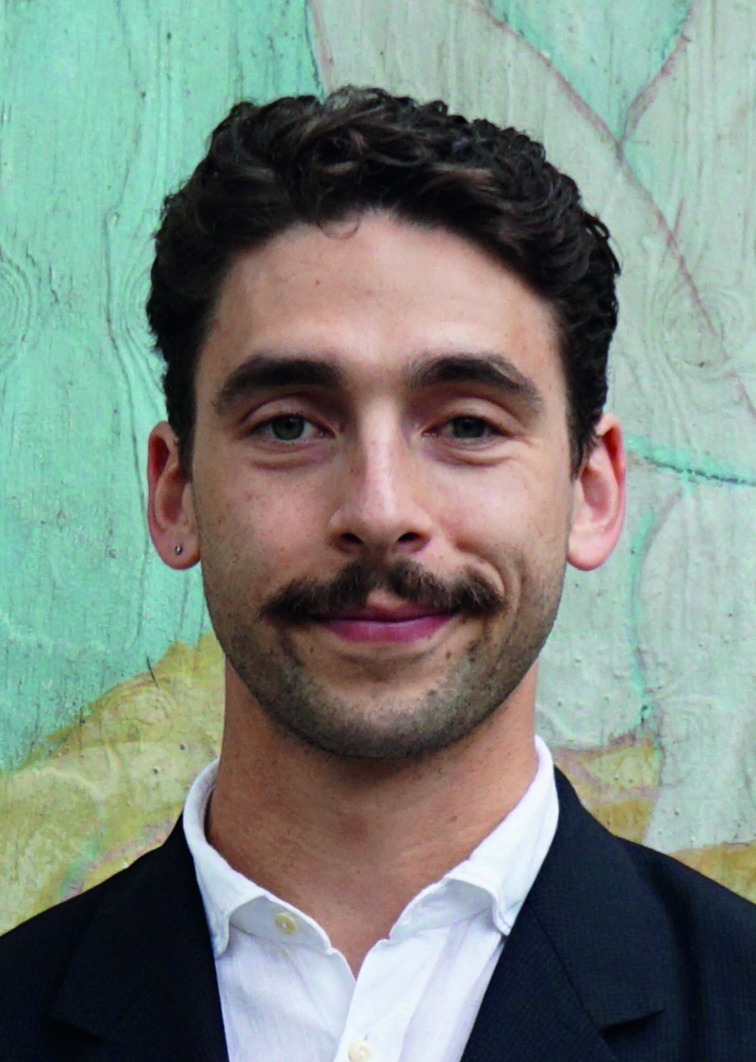

## Biographical Information

*Christopher D. Lima is a Howard Hughes Medical Institute Investigator at the Sloan Kettering Institute in New York City where he is Chair and Member of the Structural Biology Program. He holds an Alfred P. Sloan endowed chair and is a Professor in the Weill Cornell and Sloan Kettering Graduate Schools. He received his PhD from Northwestern University for resolving the structure of DNA topoisomerase I. As a Helen Hay Whitney Fellow at Columbia University, his postdoctoral studies focused on resolving mechanisms underlying nucleotidyl transferases. He joined the faculty at Weill Cornell in 1998 and moved his lab to Sloan Kettering in 2003. His research investigates quality control pathways related to RNA processing and post‐translational modification by ubiquitin and ubiquitin‐like proteins*.

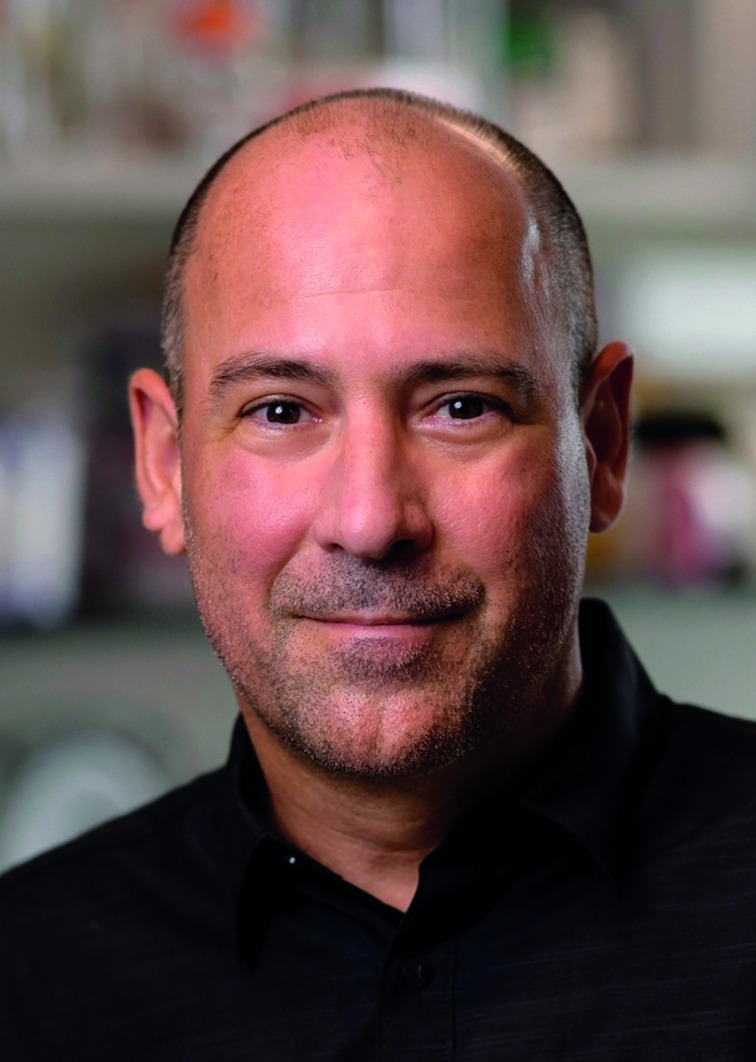

## Data Availability

Data sharing is not applicable to this article as no new data were created or analyzed in this study.

## References

[1] D. Komander , M. Rape , Annu. Rev. Biochem. 2012, 81, 203–229. 22524316 10.1146/annurev-biochem-060310-170328

[2] I. Dikic , B. A. Schulman , Nat. Rev. Mol. Cell Biol. 2023, 24, 273–287. 36284179 10.1038/s41580-022-00543-1 PMC9595094

[3] B. A. Schulman , J. Wade Harper , Nat. Rev. Mol. Cell Biol. 2009, 10, 319–331. 19352404 10.1038/nrm2673 PMC2712597

[4] L. Cappadocia , C. D. Lima , Chem. Rev. 2018, 118, 889–918. 28234446 10.1021/acs.chemrev.6b00737 PMC5815371

[5] N. Zheng , N. Shabek , Annu. Rev. Biochem. 2017, 86, 129–157. 28375744 10.1146/annurev-biochem-060815-014922

[6] L. Buetow , D. T. Huang , Nat. Rev. Mol. Cell Biol. 2016, 17, 626–642. 27485899 10.1038/nrm.2016.91 PMC6211636

[7] Y. Wang , D. Argiles-Castillo , E. I. Kane , A. Zhou , D. E. Spratt , J. Cell Sci.133 2020, DOI: 10.1242/jcs.228072.

[8] T. R. Cotton , B. C. Lechtenberg , Biochem. Soc. Trans. 2020, 48, 1737–1750. 32677670 10.1042/BST20200237 PMC7458406

[9] K. Pao , N. T. Wood , A. Knebel , K. Rafie , M. Stanley , P. D. Mabbitt , R. Sundaramoorthy , K. Hofmann , D. M. F. van Aalten , S. Virdee , Nature 2018, 556, 381–385. 29643511 10.1038/s41586-018-0026-1

[10] R. Yau , M. Rape , Nat. Cell Biol. 2016, 18, 579–586. 27230526 10.1038/ncb3358

[11] D. A. Pérez Berrocal , K. F. Witting , H. Ovaa , M. P. C. Mulder , Front. Chem. 2020, 7, 1–9. 10.3389/fchem.2019.00931 PMC6987259 32039151

[12] J. A. Ronau , J. F. Beckmann , M. Hochstrasser , Cell Res. 2016, 26, 441–456. 27012468 10.1038/cr.2016.38 PMC4822132

[13] V. L. Schramm , Annu. Rev. Biochem. 2011, 80, 703–32. 21675920 10.1146/annurev-biochem-061809-100742 PMC5502542

[14] H. Ovaa , Nat. Rev. Cancer 2007, 7, 613–620. 17646866 10.1038/nrc2128

[15] C. M. Pickart , I. A. Rose , J. Biol. Chem. 1986, 261, 10210–10217. 3015923

[16] A. Borodovsky , B. M. Kessler , R. Casagrande , H. S. Overkleeft , K. D. Wilkinson , H. L. Ploegh , EMBO J. 2001, 20, 5187–5196. 11566882 10.1093/emboj/20.18.5187 PMC125629

[17] A. Borodovsky , H. Ovaa , N. Kolli , T. Gan-Erdene , K. D. Wilkinson , H. L. Ploegh , B. M. Kessler , Chem. Biol. 2002, 9, 1149–1159. 12401499 10.1016/s1074-5521(02)00248-x

[18] J. Hemelaar , A. Borodovsky , B. M. Kessler , D. Reverter , J. Cook , N. Kolli , T. Gan-Erdene , K. D. Wilkinson , G. Gill , C. D. Lima , H. L. Ploegh , H. Ovaa , Mol. Cell. Biol. 2004, 24, 84–95. 14673145 10.1128/MCB.24.1.84-95.2004 PMC303361

[19] K. R. Love , R. K. Pandya , E. Spooner , H. L. Ploegh , ACS Chem. Biol. 2009, 4, 275–287. 19256548 10.1021/cb9000348 PMC2693349

[20] R. Ekkebus , S. I. van Kasteren , Y. Kulathu , A. Scholten , I. Berlin , P. P. Geurink , A. de Jong , S. Goerdayal , J. Neefjes , A. J. R. Heck , D. Komander , H. Ovaa , J. Am. Chem. Soc. 2013, 135, 2867–2870. 23387960 10.1021/ja309802n PMC3585465

[21] R. Byrne , T. Mund , J. D. F. Licchesi , ChemBioChem 2017, 18, 1415–1427. 28425671 10.1002/cbic.201700006 PMC5575557

[22] M. Stanley , C. Han , A. Knebel , P. Murphy , N. Shpiro , S. Virdee , ACS Chem. Biol. 2015, 10, 1542–1554. 25845023 10.1021/acschembio.5b00118

[23] R. M. Nair , A. Seenivasan , B. Liu , D. Chen , E. D. Lowe , S. Lorenz , ACS Chem. Biol. 2021, 16, 1615–1621. 34403242 10.1021/acschembio.1c00433 PMC8453484

[24] I. R. Kelsall , D. M. Duda , J. L. Olszewski , K. Hofmann , A. Knebel , F. Langevin , N. Wood , M. Wightman , B. A. Schulman , A. F. Alpi , EMBO J. 2013, 32, 2848–2860. 24076655 10.1038/emboj.2013.209 PMC3817463

[25] D. C. Scott , D. Y. Rhee , D. M. Duda , I. R. Kelsall , J. L. Olszewski , J. A. Paulo , A. de Jong , H. Ovaa , A. F. Alpi , J. W. Harper , B. A. Schulman , Cell 2016, 166, 1198–1214.e24. 27565346 10.1016/j.cell.2016.07.027 PMC5091668

[26] D. Horn-Ghetko , D. T. Krist , J. R. Prabu , K. Baek , M. P. C. Mulder , M. Klügel , D. C. Scott , H. Ovaa , G. Kleiger , B. A. Schulman , Nature 2021, 590, 671–676. 33536622 10.1038/s41586-021-03197-9 PMC7904520

[27] L. A. Hehl , D. Horn-Ghetko , J. R. Prabu , R. Vollrath , D. T. Vu , D. A. Pérez Berrocal , M. P. C. Mulder , G. J. van der Heden van Noort , B. A. Schulman , Nat. Chem. Biol. 2024, 20, 190–200. 37620400 10.1038/s41589-023-01414-2 PMC10830417

[28] T. Wauer , D. Komander , EMBO J. 2013, 32, 2099–2112. 23727886 10.1038/emboj.2013.125 PMC3730226

[29] B. E. Riley , J. C. Lougheed , K. Callaway , M. Velasquez , E. Brecht , L. Nguyen , T. Shaler , D. Walker , Y. Yang , K. Regnstrom , L. Diep , Z. Zhang , S. Chiou , M. Bova , D. R. Artis , N. Yao , J. Baker , T. Yednock , J. A. Johnston , Nat. Commun. 2013, 4, DOI: 10.1038/ncomms2982. PMC3709503 23770887

[30] T. E. C. Condos , K. M. Dunkerley , E. A. Freeman , K. R. Barber , J. D. Aguirre , V. K. Chaugule , Y. Xiao , L. Konermann , H. Walden , G. S. Shaw , EMBO J. 2018, 37, 1–16. 30446597 10.15252/embj.2018100014 PMC6276879

[31] T. Wauer , M. Simicek , A. Schubert , D. Komander , Nature 2015, 524, 370–374. 26161729 10.1038/nature14879 PMC4984986

[32] E. M. Connelly , A. C. Rintala-Dempsey , M. Gundogdu , E. A. Freeman , J. Koszela , J. D. Aguirre , G. Zhu , O. Kämäräinen , R. Tadayon , H. Walden , G. S. Shaw , Proc. Natl. Acad. Sci. USA 2024, 121, 2017. 10.1073/pnas.2403114121 PMC11317638 39078678

[33] S. Park , D. T. Krist , A. V. Statsyuk , Chem. Sci. 2015, 6, 1770–1779. 28706640 10.1039/c4sc02340d PMC5485889

[34] D. T. Krist , S. Park , G. H. Boneh , S. E. Rice , A. V. Statsyuk , Chem. Sci. 2016, 7, 5587–5595. 27482366 10.1039/c6sc01167e PMC4965700

[35] S. Park , P. K. Foote , D. T. Krist , S. E. Rice , A. V. Statsyuk , J. Biol. Chem. 2017, 292, 16539–16553. 28710279 10.1074/jbc.M116.773200 PMC5633118

[36] D. T. Krist , P. K. Foote , A. V. Statsyuk , Curr. Protoc. Chem. Biol. 2017, 9, 11–37. 28253433 10.1002/cpch.17 PMC5335898

[37] M. P. C. Mulder , K. Witting , I. Berlin , J. N. Pruneda , K.-P. Wu , J.-G. Chang , R. Merkx , J. Bialas , M. Groettrup , A. C. O. Vertegaal , B. A. Schulman , D. Komander , J. Neefjes , F. El Oualid , H. Ovaa , Nat. Chem. Biol. 2016, 12, 523–530. 27182664 10.1038/nchembio.2084 PMC5108872

[38] K. F. Witting , G. J. van der Heden van Noort , C. Kofoed , C. Talavera Ormeño , D. el Atmioui , M. P. C. Mulder , H. Ovaa , Angew. Chemie – Int. Ed. 2018, 57, 14164–14168. 10.1002/anie.201809232 PMC6220884 30188611

[39] Y. Qiao , G. Yu , S. Z. Leeuwon , W. R. Liu , Molecules 2021, 26, 1–14. 10.3390/molecules26092619 PMC8125731 33947165

[40] C. Li , T. Wang , L. Liang , G. Chu , J. Zhang , W. He , L. Liu , J. Li , Sci. China Chem. 2023, 66, 837–844. 36684644 10.1007/s11426-022-1455-x PMC9840423

[41] A. L. Haas , I. A. Rose , J. Biol. Chem. 1982, 257, 10329–10337. 6286650

[42] A. L. Haas , J. V. Warms , A. Hershko , I. A. Rose , J. Biol. Chem. 1982, 257, 2543–2548. 6277905

[43] C. M. Pickart , E. M. Kasperek , R. Beal , A. Kim , J. Biol. Chem. 1994, 269, 7115–7123. 8125920

[44] K. D. Wilkinson , S. E. Smith , L. O'Connor , E. Sternberg , J. J. Taggart , D. A. Berges , T. Butt , Biochemistry 1990, 29, 7373–7380. 2171643 10.1021/bi00484a004

[45] Z. S. Hann , C. Ji , S. K. Olsen , X. Lu , M. C. Lux , D. S. Tan , C. D. Lima , Proc. Natl. Acad. Sci. USA 2019, 116, 15475–15484. 31235585 10.1073/pnas.1905488116 PMC6681703

[46] A. M. V. Delos Reyes , M. C. Lux , Z. S. Hann , C. Ji , T. Kochańczyk , M. DiBello , C. D. Lima , D. S. Tan , Org. Lett. 2024, 26, 4594–4599. 38781175 10.1021/acs.orglett.4c01102 PMC11165569

[47] Y. Yang , J. Kitagaki , R.-M. Dai , Y. C. Tsai , K. L. Lorick , R. L. Ludwig , S. A. Pierre , J. P. Jensen , I. V. Davydov , P. Oberoi , C.-C. H. Li , J. H. Kenten , J. A. Beutler , K. H. Vousden , A. M. Weissman , Cancer Res. 2007, 67, 9472–9481. 17909057 10.1158/0008-5472.CAN-07-0568

[48] T. A. Soucy , P. G. Smith , M. A. Milhollen , A. J. Berger , J. M. Gavin , S. Adhikari , J. E. Brownell , K. E. Burke , D. P. Cardin , S. Critchley , C. A. Cullis , A. Doucette , J. J. Garnsey , J. L. Gaulin , R. E. Gershman , A. R. Lublinsky , A. McDonald , H. Mizutani , U. Narayanan , E. J. Olhava , S. Peluso , M. Rezaei , M. D. Sintchak , T. Talreja , M. P. Thomas , T. Traore , S. Vyskocil , G. S. Weatherhead , J. Yu , J. Zhang , L. R. Dick , C. F. Claiborne , M. Rolfe , J. B. Bolen , S. P. Langston , Nature 2009, 458, 732–736. 19360080 10.1038/nature07884

[49] J. E. Brownell , M. D. Sintchak , J. M. Gavin , H. Liao , F. J. Bruzzese , N. J. Bump , T. A. Soucy , M. A. Milhollen , X. Yang , A. L. Burkhardt , J. Ma , H. K. Loke , T. Lingaraj , D. Wu , K. B. Hamman , J. J. Spelman , C. A. Cullis , S. P. Langston , S. Vyskocil , T. B. Sells , W. D. Mallender , I. Visiers , P. Li , C. F. Claiborne , M. Rolfe , J. B. Bolen , L. R. Dick , Mol. Cell 2010, 37, 102–111. 20129059 10.1016/j.molcel.2009.12.024

[50] X. He , J. Riceberg , T. Soucy , E. Koenig , J. Minissale , M. Gallery , H. Bernard , X. Yang , H. Liao , C. Rabino , P. Shah , K. Xega , Z. Yan , M. Sintchak , J. Bradley , H. Xu , M. Duffey , D. England , H. Mizutani , Z. Hu , J. Guo , R. Chau , L. R. Dick , J. E. Brownell , J. Newcomb , S. Langston , E. S. Lightcap , N. Bence , S. M. Pulukuri , Nat. Chem. Biol. 2017, 13, 1164–1171. 28892090 10.1038/nchembio.2463

[51] M. L. Hyer , M. A. Milhollen , J. Ciavarri , P. Fleming , T. Traore , D. Sappal , J. Huck , J. Shi , J. Gavin , J. Brownell , Y. Yang , B. Stringer , R. Griffin , F. Bruzzese , T. Soucy , J. Duffy , C. Rabino , J. Riceberg , K. Hoar , A. Lublinsky , S. Menon , M. Sintchak , N. Bump , S. M. Pulukuri , S. Langston , S. Tirrell , M. Kuranda , P. Veiby , J. Newcomb , P. Li , J. T. Wu , J. Powe , L. R. Dick , P. Greenspan , K. Galvin , M. Manfredi , C. Claiborne , B. S. Amidon , N. F. Bence , Nat. Med. 2018, 24, 186–193. 29334375 10.1038/nm.4474

[52] M. Misra , M. Kuhn , M. Löbel , H. An , A. V. Statsyuk , C. Sotriffer , H. Schindelin , Structure 2017, 25, 1120–1129.e3. 28578874 10.1016/j.str.2017.05.001

[53] H. An , A. V. Statsyuk , Chem. Sci. 2015, 6, 5235–5245. 28717502 10.1039/c5sc01351h PMC5500945

[54] H. An , A. V. Statsyuk , J. Am. Chem. Soc. 2013, 135, 16948–16962. 24138456 10.1021/ja4099643

[55] X. Lu , S. K. Olsen , A. D. Capili , J. S. Cisar , C. D. Lima , D. S. Tan , J. Am. Chem. Soc. 2010, 132, 1748–1749. 20099854 10.1021/ja9088549 PMC2830896

[56] S. K. Olsen , A. D. Capili , X. Lu , D. S. Tan , C. D. Lima , Nature 2010, 463, 906–912. 20164921 10.1038/nature08765 PMC2866016

[57] H. An , A. V. Statsyuk , Chem. Commun. 2016, 52, 2477–2480. 10.1039/c5cc08592f 26575161

[58] P. Sung , S. Prakash , L. Prakash , J. Mol. Biol. 1991, 221, 745–749. 1658333 10.1016/0022-2836(91)80169-u

[59] M. L. Sullivan , R. D. Vierstra , J. Biol. Chem. 1993, 268, 8777–8780. 8386169

[60] T. Miura , W. Klaus , B. Gsell , C. Miyamoto , H. Senn , J. Mol. Biol. 1999, 290, 213–228. 10388568 10.1006/jmbi.1999.2859

[61] M. J. Eddins , C. M. Carlile , K. M. Gomez , C. M. Pickart , C. Wolberger , Nat. Struct. Mol. Biol. 2006, 13, 915–920. 16980971 10.1038/nsmb1148

[62] H. B. Kamadurai , J. Souphron , D. C. Scott , D. M. Duda , D. J. Miller , D. Stringer , R. C. Piper , B. A. Schulman , Mol. Cell 2009, 36, 1095–1102. 20064473 10.1016/j.molcel.2009.11.010 PMC2859195

[63] J. N. Pruneda , F. D. Smith , A. Daurie , D. L. Swaney , J. Villén , J. D. Scott , A. W. Stadnyk , I. Le Trong , R. E. Stenkamp , R. E. Klevit , J. R. Rohde , P. S. Brzovic , EMBO J. 2014, 33, 437–449. 24446487 10.1002/embj.201386386 PMC3989626

[64] Y.-S. Choi , Y.-J. Lee , S.-Y. Lee , L. Shi , J.-H. Ha , H.-K. Cheong , C. Cheong , R. E. Cohen , K.-S. Ryu , J. Biol. Chem. 2015, 290, 2251–2263. 25471371 10.1074/jbc.M114.624809 PMC4303676

[65] D. C. Scott , V. O. Sviderskiy , J. K. Monda , J. R. Lydeard , S. E. Cho , J. W. Harper , B. A. Schulman , Cell 2014, 157, 1671–1684. 24949976 10.1016/j.cell.2014.04.037 PMC4247792

[66] R. Wiener , X. Zhang , T. Wang , C. Wolberger , Nature 2012, 483, 618–622. 22367539 10.1038/nature10911 PMC3319311

[67] H. Dou , L. Buetow , G. J. Sibbet , K. Cameron , D. T. Huang , Nat. Struct. Mol. Biol. 2012, 19, 876–883. 22902369 10.1038/nsmb.2379 PMC3880866

[68] V. P. Ronchi , J. M. Klein , A. L. Haas , J. Biol. Chem. 2013, 288, 10349–10360. 23439649 10.1074/jbc.M113.458059 PMC3624418

[69] K. Tomanov , C. Hardtke , R. Budhiraja , R. Hermkes , G. Coupland , A. Bachmair , J. Integr. Plant Biol. 2013, 55, 75–82. 23206124 10.1111/jipb.12016

[70] A. F. A. Keszei , F. Sicheri , Proc. Natl. Acad. Sci. USA 2017, 114, 1311–1316. 28115697 10.1073/pnas.1611595114 PMC5307447

[71] K. K. Dove , J. L. Olszewski , L. Martino , D. M. Duda , X. S. Wu , D. J. Miller , K. H. Reiter , K. Rittinger , B. A. Schulman , R. E. Klevit , Structure 2017, 25, 890–900.e5. 28552575 10.1016/j.str.2017.04.013 PMC5462532

[72] K. Tomanov , L. Nehlin , I. Ziba , A. Bachmair , Biochem. J. 2018, 475, 61–74. 29133528 10.1042/BCJ20170472 PMC5748838

[73] E. Sakata , T. Satoh , S. Yamamoto , Y. Yamaguchi , M. Yagi-Utsumi , E. Kurimoto , K. Tanaka , S. Wakatsuki , K. Kato , Structure 2010, 18, 138–147. 20152160 10.1016/j.str.2009.11.007

[74] D. J. Wasilko , Q. Huang , Y. Mao , Elife 2018, 7, 1–17. 10.7554/eLife.36154 PMC6063727 30015617

[75] P. A. DaRosa , J. S. Harrison , A. Zelter , T. N. Davis , P. Brzovic , B. Kuhlman , R. E. Klevit , Mol. Cell 2018, 72, 753–765.e6. 30392931 10.1016/j.molcel.2018.09.029 PMC6239910

[76] J. F. de Oliveira , P. F. V. do Prado , S. S. da Costa , M. L. Sforça , C. Canateli , A. T. Ranzani , M. Maschietto , P. S. L. de Oliveira , P. A. Otto , R. E. Klevit , A. C. V. Krepischi , C. Rosenberg , K. G. Franchini , Nat. Chem. Biol. 2019, 15, 62–70. 30531907 10.1038/s41589-018-0177-2 PMC6626659

[77] M. Anandapadamanaban , N. C. Kyriakidis , V. Csizmók , A. Wallenhammar , A. C. Espinosa , A. Ahlner , A. R. Round , J. Trewhella , M. Moche , M. Wahren-Herlenius , M. Sunnerhagen , J. Biol. Chem. 2019, 294, 11404–11419. 31160341 10.1074/jbc.RA119.008485 PMC6663867

[78] M. Lee , S. Lee , J. Choi , M. Ryu , M. Lee , J. Kim , E. Hwang , C. Lee , S. Chi , K. Ryu , FEBS J. 2022, 289, 3568–3586. 35048531 10.1111/febs.16360 PMC9304225

[79] J. J. Peter , H. M. Magnussen , P. A. DaRosa , D. Millrine , S. P. Matthews , F. Lamoliatte , R. Sundaramoorthy , R. R. Kopito , Y. Kulathu , EMBO J. 2022, 41, e111015. 36121123 10.15252/embj.2022111015 PMC9627666

[80] M. Birkou , G. N. Delegkou , K. D. Marousis , N. Fragkaki , T. Toro , V. Episkopou , G. A. Spyroulias , Int. J. Mol. Sci. 2022, 23, 10585. 36142504 10.3390/ijms231810585 PMC9501438

[81] K. E. Wickliffe , S. Lorenz , D. E. Wemmer , J. Kuriyan , M. Rape , Cell 2011, 144, 769–781. 21376237 10.1016/j.cell.2011.01.035 PMC3072108

[82] R. C. Page , J. N. Pruneda , J. Amick , R. E. Klevit , S. Misra , Biochemistry 2012, 51, 4175–4187. 22551455 10.1021/bi300058m PMC3366460

[83] J. N. Pruneda , K. E. Stoll , L. J. Bolton , P. S. Brzovic , R. E. Klevit , Biochemistry 2011, 50, 1624–1633. 21226485 10.1021/bi101913m PMC3056393

[84] J. N. Pruneda , P. J. Littlefield , S. E. Soss , K. A. Nordquist , W. J. Chazin , P. S. Brzovic , R. E. Klevit , Mol. Cell 2012, 47, 933–942. 22885007 10.1016/j.molcel.2012.07.001 PMC3462262

[85] Y.-C. Juang , M.-C. Landry , M. Sanches , V. Vittal , C. C. Y. Leung , D. F. Ceccarelli , A.-R. F. Mateo , J. N. Pruneda , D. Y. L. Mao , R. K. Szilard , S. Orlicky , M. Munro , P. S. Brzovic , R. E. Klevit , F. Sicheri , D. Durocher , Mol. Cell 2012, 45, 384–397. 22325355 10.1016/j.molcel.2012.01.011 PMC3306812

[86] S. E. Soss , R. E. Klevit , W. J. Chazin , Biochemistry 2013, 52, 2991–2999. 23550736 10.1021/bi3015949 PMC3666176

[87] R. Wiener , A. T. DiBello , P. M. Lombardi , C. M. Guzzo , X. Zhang , M. J. Matunis , C. Wolberger , Nat. Struct. Mol. Biol. 2013, 20, 1033–9. 23955022 10.1038/nsmb.2655 PMC3941643

[88] A. Plechanovová , E. G. Jaffray , M. H. Tatham , J. H. Naismith , R. T. Hay , Nature 2012, 489, 115–120. 22842904 10.1038/nature11376 PMC3442243

[89] L. Cappadocia , A. Pichler , C. D. Lima , Nat. Struct. Mol. Biol. 2015, 22, 968–975. 26524494 10.1038/nsmb.3116 PMC4709122

[90] L. Chang , Z. Zhang , J. Yang , S. H. McLaughlin , D. Barford , Nature 2015, 522, 450–454. 26083744 10.1038/nature14471 PMC4608048

[91] A. J. Middleton , C. L. Day , Sci. Rep. 2015, 5, 16793. 26592444 10.1038/srep16793 PMC4655369

[92] J. G. Sanchez , J. J. Chiang , K. M. J. Sparrer , S. L. Alam , M. Chi , M. D. Roganowicz , B. Sankaran , M. U. Gack , O. Pornillos , Cell Rep. 2016, 16, 1315–1325. 27425606 10.1016/j.celrep.2016.06.070 PMC5076470

[93] C. D. Hodge , I. H. Ismail , R. A. Edwards , G. L. Hura , A. T. Xiao , J. A. Tainer , M. J. Hendzel , J. N. M. Glover , J. Biol. Chem. 2016, 291, 9396–9410. 26903517 10.1074/jbc.M116.715698 PMC4850281

[94] M. G. Koliopoulos , D. Esposito , E. Christodoulou , I. A. Taylor , K. Rittinger , EMBO J. 2016, 35, 1204–1218. 27154206 10.15252/embj.201593741 PMC4864278

[95] J. D. Wright , P. D. Mace , C. L. Day , Nat. Struct. Mol. Biol. 2016, 23, 45–52. 26656854 10.1038/nsmb.3142

[96] B. C. Lechtenberg , A. Rajput , R. Sanishvili , M. K. Dobaczewska , C. F. Ware , P. D. Mace , S. J. Riedl , Nature 2016, 529, 546–50. 26789245 10.1038/nature16511 PMC4856479

[97] C. Wloka , V. Van Meervelt , D. van Gelder , N. Danda , N. Jager , C. P. Williams , G. Maglia , ACS Nano 2017, 11, 4387–4394. 28353339 10.1021/acsnano.6b07760 PMC5444049

[98] K. Nomura , M. Klejnot , D. Kowalczyk , A. K. Hock , G. J. Sibbet , K. H. Vousden , D. T. Huang , Nat. Struct. Mol. Biol. 2017, 24, 578–587. 28553961 10.1038/nsmb.3414 PMC6205632

[99] D. M. Dawidziak , J. G. Sanchez , J. M. Wagner , B. K. Ganser-Pornillos , O. Pornillos , Proteins Struct. Funct. Bioinforma. 2017, 85, 1957–1961. 10.1002/prot.25348 PMC5638660 28681414

[100] L. Yuan , Z. Lv , J. H. Atkison , S. K. Olsen , Nat. Commun. 2017, 8, 211. 28790309 10.1038/s41467-017-00272-6 PMC5548887

[101] F. E. Morreale , A. Testa , V. K. Chaugule , A. Bortoluzzi , A. Ciulli , H. Walden , J. Med. Chem. 2017, 60, 8183–8191. 28933844 10.1021/acs.jmedchem.7b01071 PMC5663392

[102] M. Gabrielsen , L. Buetow , M. A. Nakasone , S. F. Ahmed , G. J. Sibbet , B. O. Smith , W. Zhang , S. S. Sidhu , D. T. Huang , Mol. Cell 2017, 68, 456–470.e10. 29053960 10.1016/j.molcel.2017.09.027 PMC5655547

[103] A. J. Middleton , R. Budhidarmo , A. Das , J. Zhu , M. Foglizzo , P. D. Mace , C. L. Day , Nat. Commun. 2017, 8, 1788. 29176576 10.1038/s41467-017-01665-3 PMC5702613

[104] L. Martino , N. R. Brown , L. Masino , D. Esposito , K. Rittinger , Sci. Rep. 2018, 8, 68. 29311602 10.1038/s41598-017-18513-5 PMC5758712

[105] A. Patel , G. J. Sibbet , D. T. Huang , J. Biol. Chem. 2019, 294, 1240–1249. 30523153 10.1074/jbc.RA118.006045 PMC6349121

[106] R. V. Stevens , D. Esposito , K. Rittinger , Life Sci. Alliance 2019, 2, 1–14. 10.26508/lsa.201900295 PMC6487577 31028095

[107] K. M. Williams , S. Qie , J. H. Atkison , S. Salazar-Arango , J. Alan Diehl , S. K. Olsen , Nat. Commun. 2019, 10, 3296. 31341161 10.1038/s41467-019-11061-8 PMC6656757

[108] L. Kiss , J. Zeng , C. F. Dickson , D. L. Mallery , J.-C. Yang , S. H. McLaughlin , A. Boland , D. Neuhaus , L. C. James , Nat. Commun. 2019, 10, 4502. 31582740 10.1038/s41467-019-12388-y PMC6776665

[109] H. M. Magnussen , S. F. Ahmed , G. J. Sibbet , V. A. Hristova , K. Nomura , A. K. Hock , L. J. Archibald , A. G. Jamieson , D. Fushman , K. H. Vousden , A. M. Weissman , D. T. Huang , Nat. Commun. 2020, 11, 2094. 32350255 10.1038/s41467-020-15783-y PMC7190642

[110] C. Chatrin , M. Gabrielsen , L. Buetow , M. A. Nakasone , S. F. Ahmed , D. Sumpton , G. J. Sibbet , B. O. Smith , D. T. Huang , Sci. Adv. 2020, 6, DOI: 10.1126/sciadv.abc0418. PMC7500938 32948590

[111] L. Kiss , D. Clift , N. Renner , D. Neuhaus , L. C. James , Nat. Commun. 2021, 12, 1220. 33619271 10.1038/s41467-021-21443-6 PMC7900206

[112] S. Wang , R. Wang , C. Peralta , A. Yaseen , N. P. Pavletich , Nat. Struct. Mol. Biol. 2021, 28, 300–309. 33686268 10.1038/s41594-021-00568-8 PMC8378520

[113] K. A. Welsh , D. L. Bolhuis , A. E. Nederstigt , J. Boyer , B. R. S. Temple , T. Bonacci , L. Gu , A. Ordureau , J. W. Harper , J. P. Steimel , Q. Zhang , M. J. Emanuele , J. S. Harrison , N. G. Brown , EMBO J. 2022, 41, e108823. 34942047 10.15252/embj.2021108823 PMC8804933

[114] M. A. Nakasone , K. A. Majorek , M. Gabrielsen , G. J. Sibbet , B. O. Smith , D. T. Huang , Nat. Chem. Biol. 2022, 18, 422–431. 35027744 10.1038/s41589-021-00952-x PMC8964413

[115] A. Paluda , A. J. Middleton , C. Rossig , P. D. Mace , C. L. Day , Nat. Commun. 2022, 13, 1181. 35246518 10.1038/s41467-022-28782-y PMC8897509

[116] D. Kowalczyk , M. A. Nakasone , B. O. Smith , D. T. Huang , Life Sci. Alliance 2022, 5, e202201472. 35944929 10.26508/lsa.202201472 PMC9366199

[117] Z. Pietras , A. P. Duff , V. Morad , K. Wood , C. M. Jeffries , M. Sunnerhagen , Eur. Biophys. J. 2022, 51, 569–577. 36289080 10.1007/s00249-022-01620-1 PMC9675693

[118] D. Esposito , J. Dudley-Fraser , A. Garza-Garcia , K. Rittinger , Nat. Commun. 2022, 13, 7583. 36481767 10.1038/s41467-022-35300-7 PMC9732051

[119] X. S. Wang , T. R. Cotton , S. J. Trevelyan , L. W. Richardson , W. T. Lee , J. Silke , B. C. Lechtenberg , Nat. Commun. 2023, 14, 168. 36631489 10.1038/s41467-023-35871-z PMC9834252

[120] M. Pabis , M. Termathe , K. E. Ravichandran , S. D. Kienast , R. Krutyhołowa , M. Sokołowski , U. Jankowska , P. Grudnik , S. A. Leidel , S. Glatt , EMBO J. 2020, 39, e105087. 32901956 10.15252/embj.2020105087 PMC7527816

[121] H. Dou , L. Buetow , G. J. Sibbet , K. Cameron , D. T. Huang , Nat. Struct. Mol. Biol. 2013, 20, 982–986. 23851457 10.1038/nsmb.2621 PMC4471106

[122] L. Buetow , M. Gabrielsen , N. G. Anthony , H. Dou , A. Patel , H. Aitkenhead , G. J. Sibbet , B. O. Smith , D. T. Huang , Mol. Cell 2015, 58, 297–310. 25801170 10.1016/j.molcel.2015.02.017

[123] E. Branigan , A. Plechanovová , E. G. Jaffray , J. H. Naismith , R. T. Hay , Nat. Struct. Mol. Biol. 2015, 22, 597–602. 26148049 10.1038/nsmb.3052 PMC4529489

[124] P. Kumar , P. Magala , K. R. Geiger-Schuller , A. Majumdar , J. R. Tolman , C. Wolberger , Nucleic Acids Res. 2015, 43, 9039–9050. 26286193 10.1093/nar/gkv845 PMC4605308

[125] N. Merkley , K. R. Barber , G. S. Shaw , J. Biol. Chem. 2005, 280, 31732–31738. 16014632 10.1074/jbc.M505205200

[126] S. A. Serniwka , G. S. Shaw , Biochemistry 2009, 48, 12169–12179. 19928833 10.1021/bi901686j

[127] A. M. Grishin , T. E. C. Condos , K. R. Barber , F.-X. Campbell-Valois , C. Parsot , G. S. Shaw , M. Cygler , Structure 2014, 22, 878–888. 24856362 10.1016/j.str.2014.04.010

[128] S. Lorenz , M. Bhattacharyya , C. Feiler , M. Rape , J. Kuriyan , PLoS One 2016, 11, e0147550. 26828794 10.1371/journal.pone.0147550 PMC4734694

[129] C. Purbeck , Z. M. Eletr , B. Kuhlman , Biochemistry 2010, 49, 1361–1363. 20039703 10.1021/bi9014693

[130] C. Lips , T. Ritterhoff , A. Weber , M. K. Janowska , M. Mustroph , T. Sommer , R. E. Klevit , EMBO J. 2020, 39, 1–20. 10.15252/embj.2020104863 PMC7667886 33015833

[131] L. K. Ries , B. Sander , K. K. Deol , M.-A. Letzelter , E. R. Strieter , S. Lorenz , J. Biol. Chem. 2019, 294, 6113–6129. 30737286 10.1074/jbc.RA118.007014 PMC6463701

[132] M. Pan , Q. Zheng , T. Wang , L. Liang , J. Mao , C. Zuo , R. Ding , H. Ai , Y. Xie , D. Si , Y. Yu , L. Liu , M. Zhao , Nature 2021, 600, 334–338. 34789879 10.1038/s41586-021-04097-8 PMC8798225

[133] L. Yin , B. Krantz , N. S. Russell , S. Deshpande , K. D. Wilkinson , Biochemistry 2000, 39, 10001–10010. 10933821 10.1021/bi0007019

[134] P. D. Mabbitt , A. Loreto , M. A. Déry , A. J. Fletcher , M. Stanley , K. C. Pao , N. T. Wood , M. P. Coleman , S. Virdee , Nat. Chem. Biol. 2020, 16, 1227–1236. 32747811 10.1038/s41589-020-0598-6 PMC7610530

[135] K. Zhu , Z. Shan , X. Chen , Y. Cai , L. Cui , W. Yao , Z. Wang , P. Shi , C. Tian , J. Lou , Y. Xie , W. Wen , EMBO Rep. 2017, 18, 1618–1630. 28747490 10.15252/embr.201744454 PMC5579370

[136] V. K. Chaugule , L. Burchell , K. R. Barber , A. Sidhu , S. J. Leslie , G. S. Shaw , H. Walden , EMBO J. 2011, 30, 2853–2867. 21694720 10.1038/emboj.2011.204 PMC3160258

[137] J. Trempe , V. Sauvé , K. Grenier , M. Seirafi , M. Y. Tang , M. Ménade , S. Al-Abdul-Wahid , J. Krett , K. Wong , G. Kozlov , B. Nagar , E. A. Fon , K. Gehring , Science 2013, 340, 1451–1455. 23661642 10.1126/science.1237908

[138] K. H. Reiter , A. Zelter , M. K. Janowska , M. Riffle , N. Shulman , B. X. MacLean , K. Tamura , M. C. Chambers , M. J. MacCoss , T. N. Davis , M. Guttman , P. S. Brzovic , R. E. Klevit , Structure 2022, 30, 1269–1284.e6. 35716664 10.1016/j.str.2022.05.017 PMC9444911

[139] S. Wiesner , A. A. Ogunjimi , H.-R. Wang , D. Rotin , F. Sicheri , J. L. Wrana , J. D. Forman-Kay , Cell 2007, 130, 651–662. 17719543 10.1016/j.cell.2007.06.050

[140] C. Riling , H. Kamadurai , S. Kumar , C. E. O'Leary , K.-P. Wu , E. E. Manion , M. Ying , B. A. Schulman , P. M. Oliver , J. Biol. Chem. 2015, 290, 23875–23887. 26245901 10.1074/jbc.M115.649269 PMC4583040

[141] A. A. Ogunjimi , D. J. Briant , N. Pece-Barbara , C. Le Roy , G. M. Di Guglielmo , P. Kavsak , R. K. Rasmussen , B. T. Seet , F. Sicheri , J. L. Wrana , Mol. Cell 2005, 19, 297–308. 16061177 10.1016/j.molcel.2005.06.028

[142] D. T. Huang , H. W. Hunt , M. Zhuang , M. D. Ohi , J. M. Holton , B. A. Schulman , Nature 2007, 445, 394–398. 17220875 10.1038/nature05490 PMC2821831

[143] S. E. Kaiser , K. Mao , A. M. Taherbhoy , S. Yu , J. L. Olszewski , D. M. Duda , I. Kurinov , A. Deng , T. D. Fenn , D. J. Klionsky , B. A. Schulman , Nat. Struct. Mol. Biol. 2012, 19, 1242–1249. 23142976 10.1038/nsmb.2415 PMC3515690

[144] S. K. Olsen , C. D. Lima , Mol. Cell 2013, 49, 884–896. 23416107 10.1016/j.molcel.2013.01.013 PMC3625138

[145] Z. Lv , K. A. Rickman , L. Yuan , K. Williams , S. P. Selvam , A. N. Woosley , P. H. Howe , B. Ogretmen , A. Smogorzewska , S. K. Olsen , Mol. Cell 2017, 65, 699–714.e6. 28162934 10.1016/j.molcel.2017.01.008 PMC5319395

[146] I. Wallace , K. Baek , J. R. Prabu , R. Vollrath , S. von Gronau , B. A. Schulman , K. N. Swatek , Nat. Commun. 2023, 14, 7970. 38042859 10.1038/s41467-023-43711-3 PMC10693564

[147] J. W. Pierce , R. Schoenleber , G. Jesmok , J. Best , S. A. Moore , T. Collins , M. E. Gerritsen , J. Biol. Chem. 1997, 272, 21096–21103. 9261113 10.1074/jbc.272.34.21096

[148] S. Strickson , D. G. Campbell , C. H. Emmerich , A. Knebel , L. Plater , M. S. Ritorto , N. Shpiro , P. Cohen , Biochem. J. 2013, 451, 427–437. 23441730 10.1042/BJ20121651 PMC3685219

[149] D. H. Grayson , S. H. O'Donnell , Arkivoc 2002, 2003, 4–14.

[150] K. C. Pao , M. Stanley , C. Han , Y. C. Lai , P. Murphy , K. Balk , N. T. Wood , O. Corti , J. C. Corvol , M. M. K. Muqit , S. Virdee , Nat. Chem. Biol. 2016, 12, 324–331. 26928937 10.1038/nchembio.2045 PMC4909137

[151] T. Kochańczyk , Z. S. Hann , M. C. Lux , A. M. V. Delos Reyes , C. Ji , D. S. Tan , C. D. Lima , Nature 2024, 633, 216–223. 39143218 10.1038/s41586-024-07828-9 PMC11374688

[152] F. C. Streich Jr , C. D. Lima , Nature 2016, 536, 304–308. 27509863 10.1038/nature19071 PMC5019495

[153] L. Yuan , Z. Lv , M. J. Adams , S. K. Olsen , Nat. Commun. 2021, 12, 2370. 33888705 10.1038/s41467-021-22598-y PMC8062481

[154] M. Afsar , G. Liu , L. Jia , E. A. Ruben , D. Nayak , Z. Sayyad , P. S. Bury , K. E. Cano , A. Nayak , X. R. Zhao , A. Shukla , P. Sung , E. V. Wasmuth , M. U. Gack , S. K. Olsen , Nat. Commun. 2023, 14, 4786. 37553340 10.1038/s41467-023-39780-z PMC10409785

[155] G. Li , Q. Liang , P. Gong , A. H. Tencer , Z. Zhuang , Chem. Commun. (Camb). 2014, 50, 216–218. 24225431 10.1039/c3cc47382a PMC3918478

[156] L. Xu , J. Fan , Y. Wang , Z. Zhang , Y. Fu , Y.-M. Li , J. Shi , Chem. Commun. 2019, 55, 7109–7112. 10.1039/c9cc03739j 31157339

[157] L. Liang , G. Chu , Q. Qu , C. Zuo , J. Mao , Q. Zheng , J. Chen , X. Meng , Y. Jing , H. Deng , Y. Li , L. Liu , Angew. Chemie Int. Ed. 2021, 60, 17171–17177. 10.1002/anie.202105870 34021957

[158] Q. Zheng , T. Wang , J. Mao , G. Chu , L. Liang , Y. Jing , C. Zuo , Y. Yu , H. Hu , M. Pan , Nat. Protoc. 2022, DOI: 10.1038/s41596-022-00761-z. 36323865

[159] J. Mao, H. Ai, X. Wu, Q. Zheng, H. Cai, L. Liang, Z. Tong, M. Pan, L. Liu, Preprint at *bioRxiv* **2023**, 2023.05.23.542033 https://doi.org/10.1101/2023.05.23.542033 .

[160] M. Iconomou , D. N. Saunders , Biochem. J. 2016, 473, 4083–4101. 27834739 10.1042/BCJ20160719 PMC5103871

[161] L. Merino-Cacho , O. Barroso-Gomila , S. Hernández-Sánchez , J. Ramirez , U. Mayor , J. D. Sutherland , R. Barrio , ChemBioChem 2024, 25, 1–8. 10.1002/cbic.202300746 38081789

[162] H. B. Kamadurai , Y. Qiu , A. Deng , J. S. Harrison , C. MacDonald , M. Actis , P. Rodrigues , D. J. Miller , J. Souphron , S. M. Lewis , I. Kurinov , N. Fujii , M. Hammel , R. Piper , B. Kuhlman , B. A. Schulman , Elife 2013, 2, 1–26. 10.7554/eLife.00828 PMC3738095 23936628

[163] N. G. Brown , R. VanderLinden , E. R. Watson , R. Qiao , C. R. R. Grace , M. Yamaguchi , F. Weissmann , J. J. Frye , P. Dube , S. E. Cho , M. L. Actis , P. Rodrigues , N. Fujii , J. M. Peters , H. Stark , B. A. Schulman , Proc. Natl. Acad. Sci. USA 2015, 112, 5272–5279. 25825779 10.1073/pnas.1504161112 PMC4418899

[164] M. Yamaguchi , R. VanderLinden , F. Weissmann , R. Qiao , P. Dube , N. G. Brown , D. Haselbach , W. Zhang , S. S. Sidhu , J.-M. Peters , H. Stark , B. A. Schulman , Mol. Cell 2016, 63, 593–607. 27522463 10.1016/j.molcel.2016.07.003 PMC5148128

[165] N. Varejão , J. Lascorz , J. Codina-Fabra , G. Bellí , H. Borràs-Gas , J. Torres-Rosell , D. Reverter , Nat. Commun. 2021, 12, 7013. 34853311 10.1038/s41467-021-27301-9 PMC8636563

[166] S. Goffinont , F. Coste , P. Prieu-Serandon , L. Mance , V. Gaudon , N. Garnier , B. Castaing , M. J. Suskiewicz , J. Biol. Chem. 2023, 299, 1–14. 10.1016/j.jbc.2023.104870 PMC10404613 37247759

[167] K. Baek , D. T. Krist , J. R. Prabu , S. Hill , M. Klügel , L.-M. Neumaier , S. von Gronau , G. Kleiger , B. A. Schulman , Nature 2020, 578, 461–466. 32051583 10.1038/s41586-020-2000-y PMC7050210

[168] D. T. Krist , A. V. Statsyuk , Biochemistry 2015, 54, 4411–4414. 26161728 10.1021/acs.biochem.5b00625 PMC4527872

[169] Y. Zhang , T. Hirota , K. Kuwata , S. Oishi , S. G. Gramani , J. W. Bode , J. Am. Chem. Soc. 2019, 141, 14742–14751. 31436980 10.1021/jacs.9b06820

[170] S. Mathur , A. J. Fletcher , E. Branigan , R. T. Hay , S. Virdee , Cell Chem. Biol. 2020, 27, 74–82.e6. 31859248 10.1016/j.chembiol.2019.11.013 PMC6963778

[171] F. Bustos , S. Mathur , C. Espejo-Serrano , R. Toth , C. J. Hastie , S. Virdee , G. M. Findlay , Life Sci. Alliance 2022, 5, e202101248. 35764390 10.26508/lsa.202101248 PMC9240097

[172] J. Liwocha , D. T. Krist , G. J. van der Heden van Noort , F. M. Hansen , V. H. Truong , O. Karayel , N. Purser , D. Houston , N. Burton , M. J. Bostock , M. Sattler , M. Mann , J. S. Harrison , G. Kleiger , H. Ovaa , B. A. Schulman , Nat. Chem. Biol. 2021, 17, 272–279. 33288957 10.1038/s41589-020-00696-0 PMC7904580

